# Damage sensing by a Nox-Ask1-MKK3-p38 signaling pathway mediates regeneration in the adult *Drosophila* midgut

**DOI:** 10.1038/s41467-019-12336-w

**Published:** 2019-09-25

**Authors:** Parthive H. Patel, Clothilde Pénalva, Michael Kardorff, Marianne Roca, Bojana Pavlović, Anja Thiel, Aurelio A. Teleman, Bruce A. Edgar

**Affiliations:** 1 Elizabeth Blackwell Institute for Health Research, University of Bristol, Bristol, BS8 1UH UK; 2 School of Cellular and Molecular Medicine, University of Bristol, Bristol, BS8 1TD UK; 3 Huntsman Cancer Institute, University of Utah, Salt Lake City, UT 84112 USA; 4 German Cancer Research Center (DKFZ), 69120 Heidelberg, Germany; 5 Center for Molecular Biology, University of Heidelberg (ZMBH), 69120 Heidelberg, Germany

**Keywords:** Stress signalling, Intestinal stem cells, Multipotent stem cells, Regeneration, Stem-cell niche

## Abstract

Epithelia are exposed to diverse types of stress and damage from pathogens and the environment, and respond by regenerating. Yet, the proximal mechanisms that sense epithelial damage remain poorly understood. Here we report that p38 signaling is activated in adult *Drosophila* midgut enterocytes in response to diverse stresses including pathogenic bacterial infection and chemical and mechanical insult. Two upstream kinases, Ask1 and Licorne (MKK3), are required for p38 activation following infection, oxidative stress, detergent exposure and wounding. Ask1-p38 signaling in enterocytes is required upon infection to promote full intestinal stem cell (ISC) activation and regeneration, partly through Upd3/Jak-Stat signaling. Furthermore, reactive oxygen species (ROS) produced by the NADPH oxidase Nox in enterocytes, are required for p38 activation in enterocytes following infection or wounding, and for ISC activation upon infection or detergent exposure. We propose that Nox-ROS-Ask1-MKK3-p38 signaling in enterocytes integrates multiple different stresses to induce regeneration.

## Abstract (web summary)

Epithelia are exposed to diverse types of environmental stress, but the mechanisms by which epithelial cells sense stress are not well understood. Here, the authors show that a Nox-ROS-Ask1-MKK3-p38 signaling axis integrates various types of stress to promote intestinal regeneration.

## Introduction

Epithelial tissues are regularly exposed to a variety of stresses caused by pathogens, toxins, and other environmental factors. Nevertheless, barrier epithelia have an incredible ability to not only defend themselves from these insults but also to repair themselves. One exquisite example is the intestinal epithelium, which upon infection (1) defends itself against pathogens by producing mucus, reactive oxygen species (ROS), and antimicrobial peptides (AMPs), and (2) mobilizes resident intestinal stem cells (ISCs) to rapidly replace lost epithelial cells to maintain epithelial integrity^1^. While epithelia have sophisticated systems to sense tissue stress and promote defense responses, how stress sensing is achieved and then coupled to epithelial regeneration is not well understood.

Similar to the mammalian intestine, the adult *Drosophila* intestine (or midgut) is maintained by ISCs, which produce transient progeny, enteroblasts (EBs) that can differentiate into either enterocytes (ECs) or enteroendocrine cells (EEs)^2,3^. These epithelial cells grow upon a basement membrane (BM) overlaying the visceral muscle (VM), which together with the EBs, ECs, and EEs comprise the epithelial ISC niche^4^. Upon damage, intestinal regeneration in *Drosophila* is mediated by the cytokine/JAK-STAT, EGFR/Ras/MAPK, insulin, TOR, Wnt/Wg, BMP/TGFβ, PDGF/VEGF, Hh, Notch, JNK), Hpo, and Ret signaling^5,6^. The various niche cells (EBs, ECs, and VM) together provide most of these signals to regulate ISC proliferation, self-renewal, and differentiation^5^. Furthermore, the *Drosophila* ISC niche can sense pathogenic, chemical, and mechanical stresses such as *Erwinia carotovora carotovora* (*Ecc15*), *Pseudomonas entomophila* (*P.e*.), ROS (paraquat), detergents (dextran sodium sulfate, DSS), DNA damage (bleomycin), tumor growth, and EC detachment from the BM through the JNK and Hpo signaling pathways^7–12^. However, precisely how these pathways sense these diverse types of stresses and couple them to tissue regeneration is still not well understood.

Stress-activated protein kinases (SAPKs), which include the Jun-N-terminal kinase (JNK) and the p38 mitogen-activated protein kinase (MAPK), are a conserved subfamily of MAPKs that sense and elicit cellular responses to stress^13^. SAPKs are thought to promote both fly intestinal regeneration and immunity. JNK is activated in ECs by stress (i.e., ROS (paraquat), pathogenic bacterial infection (*Ecc15*, *P.e*.), tumor growth, and detachment from the BM), and increasing JNK activity in ECs is sufficient to promote ISC proliferation by stimulating cytokine (*upd2*, *upd3*) expression^8–10,14^. These cytokines facilitate regeneration by stimulating JAK-STAT activity in ISCs to promote their proliferation and in EBs to promote their differentiation^8,10,15–17^. Together, these data have led to the common view that JNK signaling can sense stress and couple it to intestinal regeneration^18^. Nevertheless, JNK was found to be only mildly required in ECs for resistance (or survival) to *Ecc15*^10^ and for *upd3* induction upon *Ecc15* infection^19^, suggesting that other pathways in addition to JNK are required to couple stress to regeneration.

On the other hand, previous work in *Caenorhabditis elegans*, the adult fly intestine, and human cultured intestinal cancer cells suggest that p38 signaling is required for sensing and providing resistance toward pathogenic bacteria^20,21^. It has been reported in the fly intestine that bacterial wall components (i.e., peptidoglycans) and metabolites (i.e., uracil) activate p38 signaling, which subsequently confers resistance by boosting ROS generation (i.e., H_2_O_2_) by upregulating the expression of the nicotinimide adenine dinucleotide phosphate (NADPH) oxidase (Nox) and Dual oxidase (Duox)^21,22^. Indeed, activated p38a, one of the three fly p38 MAPKs (p38a, p38b, and p38c), was shown to upregulate *Duox* expression through phosphorylation and activation of the activating transcription factor 2 (Atf2)^21,22^. In contrast, another *Drosophila* study found that p38a and p38b mutant flies were as sensitive to *P.e*. infection as control flies, and that p38a was not required for *Duox* expression upon infection^23^. Instead, they reported that p38c is upregulated after pathogenic bacterial infection to not only promote Duox-mediated ROS production via Atf2 but also to restrict ISC proliferation by limiting cytokine (Upd3) expression^23^. Although it is not clear which fly p38 (p38a or p38c) is involved, these data suggest that p38 signaling promotes fly intestinal immunity through Duox-mediated ROS production. However, whether p38 signaling plays an important role in *Drosophila* intestinal regeneration is not known.

Work in the mouse intestinal epithelium suggests a role for p38 signaling in intestinal regeneration; *p38α* (one of four p38s in mice and homologous to fly p38a and p38b) was required in the mouse intestinal epithelium for repair after detergent (DSS)-induced damage^24,25^. Consistent with this, p38b signaling in fly ECs was also shown to partially regulate *upd3* expression in *Ecc15*-infected flies^19^; however, its requirement for ISC response and midgut regeneration was not directly tested. Although these studies suggest a role for p38 in intestinal regeneration, how p38 is activated upon intestinal damage, which cell types utilize it, and how p38 promotes intestinal regeneration are not well understood.

ROS are also thought to promote intestinal turnover and regeneration. The NADPH oxidase, Nox, has been reported to produce ROS (O_2_^•^) upon exposure to commensal bacteria such as *Lactobacillus plantarum*, which also consequently promotes fly and mouse ISC proliferation^26^. Furthermore, paraquat or H_2_O_2_ exposure promote fly intestinal epithelial turnover^10^. In contrast, antioxidants (i.e., *N*-acetyl cysteine and glutathione) feeding or *Duox* depletion blocked intestinal regeneration in *Ecc15*-infected flies^10^. Moreover, *Nox1* knockout mice showed reduced ISC proliferation after DSS exposure^27^. Although ROS can stimulate JNK activity^14^, how ROS promote intestinal regeneration is not well understood. In addition, although it has been reported that intestinal ROS production is boosted by p38 signaling, it is not known whether ROS can activate p38 signaling to effect intestinal regeneration.

SAPKs (JNK, p38) are activated by the upstream SAP kinase kinases (SAP2Ks) such as MKK4/7 and MKK3/6, which activate JNK and p38, respectively^13^. SAP2Ks such as MKK3 are dual-specificity kinases that can phosphorylate the highly conserved dual-phosphorylation site (TGY) of p38s to further promote their activity^13,28^. The sole fly MKK3, Licorne, can phosphorylate both p38a and p38b but not p38c, which lacks the dual-phosphorylation site^29^. SAP2Ks are activated by SAP kinase kinase kinases (SAP3Ks) (i.e., MAPK/ERK kinase kinase 1 (Mekk1)^30^, Apoptosis signal-regulating kinase 1 (Ask1), transforming growth factor-β (TGFβ)-activated kinase 1 (Tak1)), which are activated by a variety of stresses, such as heat, oxidative, osmotic, or pathogen. In flies, both Mekk1 and Tak1 signaling have been reported to play important roles in sensing pathogenic bacteria and promoting gut immunity^21,23,31^. In *C. elegans*, Ask1/nsy-1 is required for pathogen resistance^20^. Ask1 is also thought to sense oxidative stress^32^ and in flies it promotes wing disc regeneration via JNK and p38^33^. A role for SAP2Ks and SAP3Ks in intestinal regeneration, however, is unknown. As ROS promote tissue regeneration in other contexts^34–36^, SAP3Ks such as Ask1 may have an important role in coupling intestinal immunity to regeneration.

Here, we show that p38 signaling promotes intestinal regeneration by sensing a variety of stresses in ECs and promoting ISC proliferation. In addition, we find that Nox-derived ROS and Ask1 are required to fully activate MKK3-p38 signaling to promote intestinal regeneration upon pathogenic infection. Furthermore, we find that Nox-derived ROS are required to promote intestinal regeneration upon detergent exposure. Together, our data suggest that Nox-Ask1-MKK3-p38 signaling senses multiple different stresses and effectively couples these stresses with regeneration.

## Results

### p38 in ECs promotes ISC-mediated regeneration upon damage

**Fig. 1:**
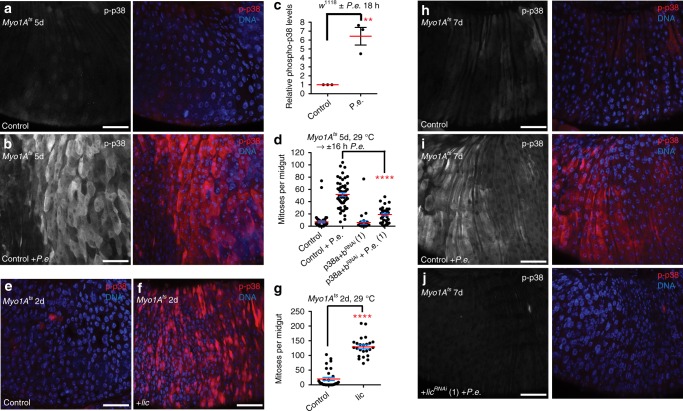
Mkk3-p38 signaling in ECs promotes ISC proliferation upon damage. **a**, **b** Both p38a and p38b (p38a + b) are activated in ECs upon *P.e*. infection. p38 activation increased in ECs of *P.e*.-infected control (EC-specific, *Myo1A*^*ts*^) midguts (**b** right, red) compared with uninfected control midguts (**a** right, red). **c** p38 is activated in the midguts upon pathogen infection. Mean (with s.e.m.) phospho-p38 levels are increased in *P.e*.-infected midguts (*n* = 3 midguts from one experiment) compared with uninfected control midguts (*n* = 3 midguts from one experiment) (unpaired *t* test (on data not normalized to control); *P* = 0.0085). **d** Both p38a and p38b are required in ECs to promote ISC proliferation upon *P.e*. infection. The ISC response is blocked in *P.e*.-infected midguts expressing *p38a* *+* *b*^*RNAi*^ in ECs for 5 days with *Myo1A*^*ts*^ compared with *P.e*.-infected control (*Myo1A*^*ts*^) midguts (Mann–Whitney; *P* < 0.0001). The mean number of phosphorylated histone H3 Ser 10-positive cells per midgut with s.e.m. in control midguts (*n* = 55 midguts), *P.e*.-infected control midguts (*n* = 53 midguts), midguts expressing *p38a* + *b*^*RNAi*^ in ECs for 5 days (*n* = 28 midguts) and *P.e*.-infected midguts expressing *p38a* + *b*^*RNAi*^ in ECs for 5 days (*n* = 32 midguts). Midguts pooled from three independent experiments. **e**, **f** Mkk3/Licorne can activate p38 in ECs. p38 activation increased in the midguts overexpressing Licorne in ECs for 2 days with *Myo1A*^*ts*^ (**f**, red) compared with control midguts (**e** red). **g** Overexpression of Mkk3/Licorne in ECs for 2 days with *Myo1A*^*ts*^ promotes ISC proliferation. The mean number of phosphorylated histone H3 Ser 10-positive cells per midgut with s.e.m. in control (*Myo1A*^*ts*^) midguts (*n* = 31 midguts) and in midguts expressing *lic* in ECs for 2 days with *Myo1A*^*ts*^ (*n* = 24 midguts) (Mann–Whitney; *p* < 0.0001). Midguts pooled from three independent experiments. **h**–**j** Mkk3/Licorne is required for p38 activation in ECs upon *P.e*. infection. p38 activation increases in ECs in *P.e*.-infected control (*Myo1A*^*ts*^) midguts (**i** right, red) compared with uninfected control midguts (**h** right, red). p38 activation is blocked however in *P.e*.-infected midguts expressing *lic*^*RNAi*^ for 7 days with *Myo1A*^*ts*^ (**j** right, red) compared with *P.e*.-infected control midguts. DNA is in blue. Scale bars in **a**, **b**, **e**, **f**, **h**–**j** are 50 μm. Representative images in **a**, **b**, **e**, **f** from three experiments; **h**–**j**, two experiments. Source data are provided as a Source Data File

Previously, p38 was described to be mostly inactive in the adult fly midgut^21^. Using an anti-phospho-p38 antibody that should recognize both activated p38a and p38b, we found that in both male and female midguts p38 was activated often in the anterior midgut (R1, anterior R2) and occasionally in (and around) the mid-midgut (R3)^37^. In the posterior midgut (R4a-R5), p38 activity was typically absent and we only occasionally observed low levels of activated p38. p38a was shown to be activated in midgut ECs after feeding of soluble microbial extract or peptidoglycan from *Ecc15*^21^. Consistent with this result, we found that p38 was strongly activated in ECs throughout the midgut after oral infection with another Gram-negative bacterium *P.e*. relative to uninfected control midguts (Fig. 1a–c). Single depletion of either p38a or p38b in ECs with UAS-*RNAi* transgenes under the control of the EC-specific driver *Myo1A-GAL4* (*Myo1A*^*ts*^) reduced neither the basal active p38 levels nor the p38 activation after *P.e*. infection relative to controls (Supplementary Fig. 2a–d). Although depletion of both p38a and p38b simultaneously (*p38a* *+* *b*) using an *RNAi* that targets both mRNAs with *Myo1A*^*ts*^ did not detectably reduce the basal levels of p38 activation, it did however suppress the p38 activation upon *P.e*. infection relative to control-infected midguts (Supplementary Fig. 1a, b). This suggested that the phospho-p38-specific antibody recognizes both the phosphorylatable forms of fly p38 (p38a and p38b) but not p38c, which lacks the phosphorylation site^29,38^. Infection by pathogenic bacteria stimulates ISC proliferation and the replacement of stressed or dying ECs^8,10^. To determine whether p38 activation after *P.e*. infection occurred in newborn or old ECs, or both, we labeled ISCs and their progeny with green fluorescent protein (GFP) using the ISC lineage tracing tool *esg-GAL4 tubGAL80*^*ts*^ *UAS-GFP; UAS-FLP, act* *<* *CDC* *>* *GAL4* (*esg*^*ts*^*F/O*)^8^. During *P.e*. infection, p38 activation was observed in both newborn and old ECs in the epithelium, but at low levels in ECs of controls (Supplementary Fig. 1c, d). These data suggest that both old and new ECs can sense stress during pathogenic bacterial infection and activate p38a + b signaling.

In addition to ECs, we observed strong p38 activation in the VM after *P.e*. infection relative to the VM of control, uninfected midguts (Supplementary Fig. 1e–f). To determine whether p38 activity in ECs and the VM was important for the ISC response to infection, we depleted both *p38a* *+* *b* in the VM with *how-GAL4, tubGAL80*^*ts*^ (*how*^*ts*^) or in ECs with *Myo1A*^*ts*^. Depletion of *p38a* *+* *b* in VM neither affected basal ISC divisions relative to uninfected control midguts nor the ISC response to *P.e*. infection relative to infected control midguts (Supplementary Fig. 1g). Although depletion of *p38a*, *p38b*, or both *p38a* *+* *b* or overexpression of p38^DN^ in ECs did not affect basal ISC divisions relative to uninfected control midguts, blocking both *p38a* *+* *b* in ECs, but neither *p38a* or *p38b* alone, did strongly suppress (by 63.2% with *RNAi* (1); 57.9%, *RNAi* (2)) the ISC response to *P.e*. infection relative to infected controls (Fig. 1d; Supplementary Figs. 1h, j and 2e–g). Interestingly, we found that the ISC response to *P.e*. infection was mildly suppressed in the midguts depleted for *p38b* in ECs (by 24.1%) or mutant for *p38b* (*p38b*^*ex9*^) (22.5% in *p38b*^*ex9*^/+; 37.2%, *p38b*^*ex9*^/*p38b*^*ex9*^)^39^ (Supplementary Fig. 2g, i). In contrast, the ISC response in *p38a* and *p38c* double-mutant (*p38a*^*1*^) midguts^23,29^ was not blocked upon *P.e*. infection (Supplementary Fig. 2h). Together, these data suggest that upon *P.e*. infection, *p38b* is required in ECs for the ISC response, and that *p38a* may be required to sense stress in the absence of *p38b* or together with *p38b*. These data suggest that p38a + b signaling is required in ECs to promote ISC-mediated regeneration.

The SAP2K, MKK3, is known to activate p38 in mammalian cells^13,28^; however, its ability to activate p38 in intestinal epithelial cells is not known. Moreover, stress responses known to be p38-dependent (i.e., osmotic stress in the larval *Drosophila* hindgut) have been previously shown to not require MKK3^40^. We found that overexpression of the *Drosophila* MKK3, *licorne* (*lic*), with the *Myo1A*^*ts*^ driver strongly activated p38 in ECs (Fig. 1e, f; Supplementary Fig. 3a–c). Furthermore, Lic overexpression in ECs for 2 days also increased ISC proliferation relative to basal ISC divisions in control midguts (Fig. 1g). Conversely, we found that while depletion of the fly MKK3, *licorne*, in ECs with *Myo1A*^*ts*^ did not noticeably affect basal active p38 levels relative to control uninfected midguts, it inhibited p38 activation upon *P.e*. infection (Fig. 1h–j, red; Supplementary Fig. 3d–i). These data suggest that p38 is activated upon infection via MKK3/Lic resulting in ISC proliferation.

**Fig. 2:**
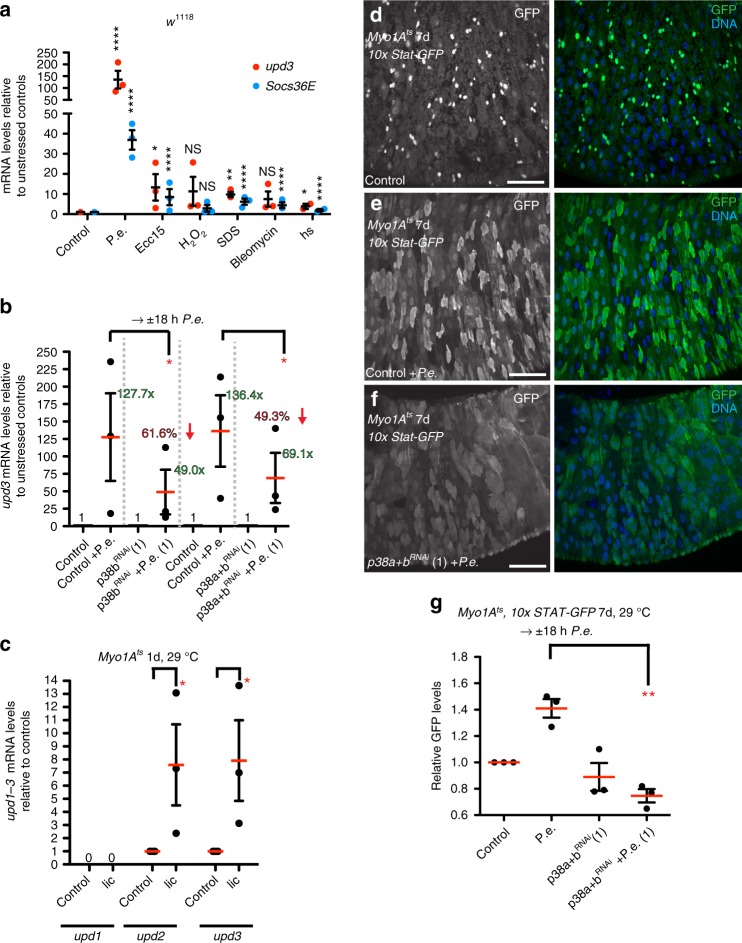
Enterocyte p38 signaling promotes ISC proliferation via cytokine Upd3/Jak-Stat signaling. **a** Multiple stresses induce cytokine and JAK-STAT target gene expression. Mean relative mRNA with s.e.m. from *n* = 3 independent experiments of cytokine upd3 mRNA levels (one-way ANOVA: *p* < 0.0001; Dunnett’s: *P.e.* < 0.0001, *Ecc15* = 0.0323, H_2_O_2_ = 0.4896, SDS = 0.0089, bleomycin = 0.409, heat shock = 0.0392) and JAK-STAT target, *Socs36E*, mRNA levels (one-way ANOVA: *p* = 0.0002; Dunnett’s: *P.e.* < 0.0001, *Ecc15* < 0.0001, H_2_O_2_ = 0.3157, SDS < 0.0001, bleomycin < 0.0001, heat shock < 0.0001) determined by qPCR from stressed and unstressed *w*^*1118*^ midguts. **b** p38 signaling promotes cytokine induction upon *P.e*. infection. Mean relative mRNA with s.e.m. from *n* = 3 independent experiments of cytokine *upd3* mRNA levels determined by qPCR from uninfected and infected midguts with *p38b-* (one-way ANOVA: *p* = 0.0223; one sample *t* test: *p* = 0.0452) and *p38a* *+* *b*-depleted (one-way ANOVA: *p* = 0.0202; one sample *t* test: *p* = 0.0421) in ECs for 7 days and control midguts (*Myo1A*^*ts*^). **c** Overexpression of MKK3/Licorne in ECs promotes cytokine expression. Mean relative mRNA with s.e.m. from *n* = 3 independent experiments of *upd2* (one sample *t* test: *p* = 0.0488) and *upd3* mRNA (one sample *t* test: *p* = 0.0467) levels determined by qPCR on midguts overexpressing Licorne for 1 day using EC-specific, *Myo1A*^*ts*^ and control midguts (*Myo1A*^*ts*^). **d**–**g** p38 signaling in enterocytes promotes Stat activity upon *P.e*. infection. Increased *10x STAT-GFP* is observed in *P.e*.-infected control midguts (**e** right, green) compared with uninfected control midguts (**d** right, green). Reduced *10x STAT-GFP* is observed however in *P.e*.-infected midguts expressing *p38a* *+* *b*^*RNAi*^ in ECs for 7 days (**f** right, green) compared with *P.e*.-infected control midguts. Quantified GFP levels are in **g** (*n* = 3 midguts from one experiment; unpaired *t* test; *p* = 0.0017). DNA is in blue. Scale bars in **d**–**f** are 50 μm. Representative images in **d**–**g** from three experiments. Source data are provided as a Source Data File

The boost in ISC proliferation after *P.e*. infection is partly due to increased mitogen production (i.e., cytokines: Upd2, Upd3) by ECs^8^. Indeed, we found that mRNA levels encoding Upd3 and the JAK-STAT target, Socs36E, were increased after a variety of stresses, including *P.e*., *Ecc15*, H_2_O_2_, Sodium dodecyl sulfate (SDS), bleomycin, and heat shock (Fig. 2a). To determine the role of p38 in this stress-dependent cytokine induction, we tested whether *upd3* mRNA was induced after *P.e*. infection in p38-depleted midguts. Using quantitative PCR (qPCR), we found that EC-targeted *RNAis* against *p38b* and *p38a* *+* *b* suppressed the induction of *upd3* mRNA following *P.e*. infection by 62 and 49%, respectively (Fig. 2b). In support of a role for p38a + b signaling in regulating cytokine production, Licorne overexpression for 24 h was sufficient to induce *upd2* and *upd3* expression (≈8-fold) (Fig. 2c). These data suggest that MKK3-p38 signaling promotes *upd3* induction upon *P.e*. infection, largely through p38b. Cytokine-induced JAK-STAT activity in the midgut can be detected using the in vivo reporter, *10x STAT-GFP*, which we found to be high in progenitors and a few ECs in uninfected controls and in uninfected controls depleted for *p38a* *+* *b* (Fig. 2d; Supplementary Fig. 1i). However, *P.e*.-infected controls showed strongly increased *10x STAT-GFP* in most ECs, whereas this was markedly decreased in *p38a* *+* *b*-depleted midguts (Fig. 2e–g). These data suggest that p38 signaling in ECs promotes ISC-mediated regeneration by boosting cytokine Upd3 expression.

We also tested if known p38 phosphorylation targets were required in ECs to promote ISC response to infection by assessing whether ISC proliferation was affected in *P.e*.-infected flies mutant for the transcriptional regulator *Atf2* (*Atf2*^*PB*^)^41^ or in flies depleted for *Atf2* in ECs, or mutant for MAPK-activated protein kinase 2 (MAPKAP-K2 or MK2)^40^. We found that basal ISC division rates were not affected in *Atf2* mutant midguts, or in midguts depleted of *Atf2* in ECs (Supplementary Fig. 4a, b, d). Furthermore, we did not find an effect on the ISC response to *P.e*. infection in *Atf2* mutant midguts, or in midguts depleted for *Atf2* in ECs (Supplementary Fig. 4a, b). Similarly, the ISC response to *P.e*. infection was not affected in the *MK2* P-element excision deletion mutant (*MK2*^*Δ43*^) when compared with its precise excision control, *MK2*^*Δ1A*^ (Supplementary Fig. 4c). These data suggest that p38 activity in stressed ECs does not promote ISC proliferation via MK2 or ATF2.

### Chemical and mechanical stress activate p38 in the midgut

To test whether stresses in addition to pathogenic bacteria can activate p38, we tested whether several other types of stress could activate p38 in midgut ECs. Exposing flies or fly cultured S2 cells to a diverse array of stresses (i.e., Gram-negative pathogenic bacteria (*Ecc15*, *P.e*.), septic injury (*Salmonella*), heat shock, dry starvation, ROS (i.e., hydrogen peroxide (H_2_O_2_), high salt (0.2 M NaCl), metal ions) has been shown to activate p38^21,29,38^; however, only enteric infection had been shown to activate p38 in the adult fly midgut^21^. We found that p38 signaling was not activated in midguts after heat shock at 37 °C for 30 min (Supplementary Fig. 5a, b). However, in vivo reporters for JNK activity (*puc-lacZ*) and cytokine *upd3* expression (*upd3-lacZ*) were activated after heat stress (Supplementary Fig. 5c–f). We also found that feeding 0.2 M NaCl or 0.4 M NaCl for 1 week (Supplementary Fig. 5g, h) or starvation (water only) did not activate p38 in midguts. These data suggest that heat, high salt stress, or starvation stress were not able to activate p38 signaling in the fly midgut.

**Fig. 3:**
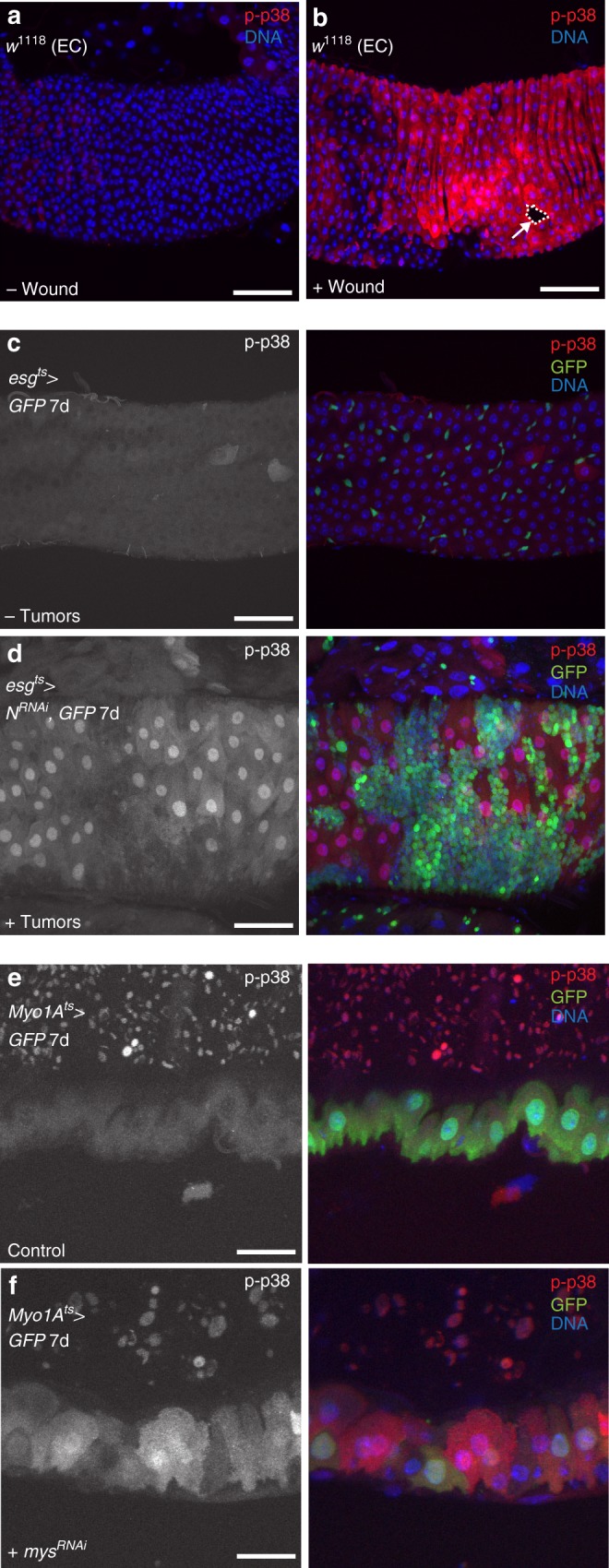
Wounding, tumor growth, and cell detachment activate p38 signaling in ECs. **a**, **b** Wounding with a needle activates p38 signaling in enterocytes (ECs) (**b** red) compared with unwounded midgut ECs (**a**). Wound site is indicated with an arrow and outlined with dashed line. **c**, **d** Tumor growth activates p38 signaling in surrounding intestinal epithelial cells. p38 signaling is activated in ECs of midguts expressing *N*^*RNAi*^ and GFP in progenitors for 7 days with *esg*^*ts*^ *>* *GFP* (**d** right, red), but not in tumor-free midguts expressing GFP alone in progenitors for 7 days with *esg*^*ts*^ *>* *GFP* (**c** right, red). **e**, **f** Intestinal epithelial cell detachment from the basement membrane activates p38 signaling. p38 signaling is increased in detaching ECs of midguts expressing *mys*^*RNAi*^ and GFP for 7 days with *Myo1A*^*ts*^ (**f** right, red) compared with midguts expressing GFP alone in ECs for 7 days with *Myo1A*^*ts*^ (**e** right, red). DNA is in blue. Scale bars in **a**–**d** are 50 μm; in **e**, **f**, 20 μm. Representative images in **a** and **b** from three experiments; **c**, **d**, **e**, **f**, two experiments

**Fig. 4:**
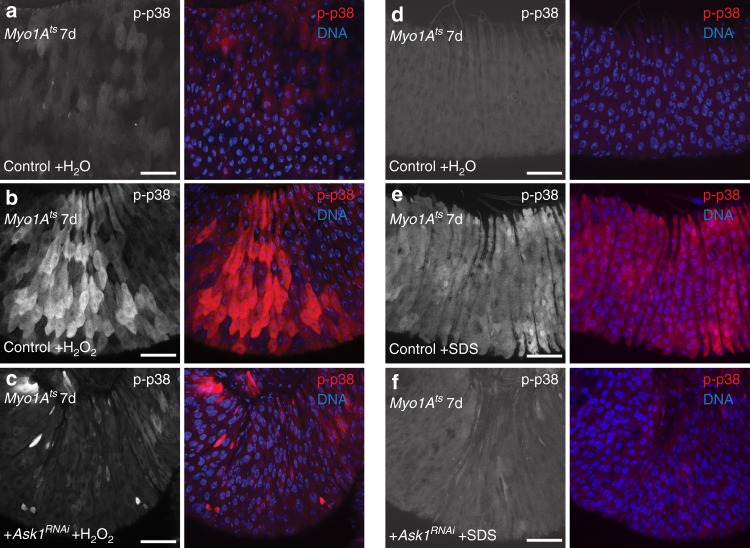
Ask1-p38 signaling is activated in ECs after ROS and detergent damage. **a**–**f** Ask1 is required for p38 activation in ECs upon H_2_O_2_ and detergent exposure. p38 activation increases in ECs in H_2_O_2_ or SDS-fed control (EC-specific, *Myo1A*^*ts*^) midguts (**b**, **e** right, red) compared with H_2_O-fed control midguts (**a**, **d** right, red). p38 activation is blocked, however, in H_2_O_2_ or SDS-fed midguts expressing *Ask1*^*RNAi*^ in ECs for 7 days with *Myo1A*^*ts*^ (**c**, **f** right, red) compared with H_2_O_2_ or SDS-fed control midguts. DNA is in blue. Scale bars in **a**–**f** are 50 μm. Representative images from three experiments

We next tested mechanical stress. Strong p38 activation was observed in the VM and ECs shortly after wounding the posterior midgut with a needle (Fig. 3a, b; Supplementary Fig. 9a, b). Interestingly, p38 was activated not only near the wound site but also in the VM (and in many ECs) throughout the midgut, suggesting propagation of stress signaling (Fig. 3b; Supplementary Fig. 9b). Previously, we reported that EC detachment by integrin subunit depletion or by rapidly growing, differentiation-defective ISC tumors activates JNK and YAP/Yki activity^9^. Similarly, we found that ISC tumor growth^9^ also activated p38 in surrounding ECs relative to control midguts bearing no tumors (Fig. 3c, d). Moreover, detaching ECs by depleting the integrin subunit βPS1 (*myospheroid*) or both αPS3 (*scab*) + αPS4 activated p38 signaling (Fig. 3e, f). Lastly, feeding flies with 1% H_2_O_2_ or 0.2% sodium dodecyl sulfate (SDS) for 2 days activated p38 in the midgut relative to midguts from flies fed water alone (Fig. 4a, b, d, e). Together these data suggest that p38 signaling responds to several different stresses, including pathogenic infection, chemical stress, and mechanical stress.

### Ask1 activates p38 upon stress and promotes regeneration

**Fig. 5:**
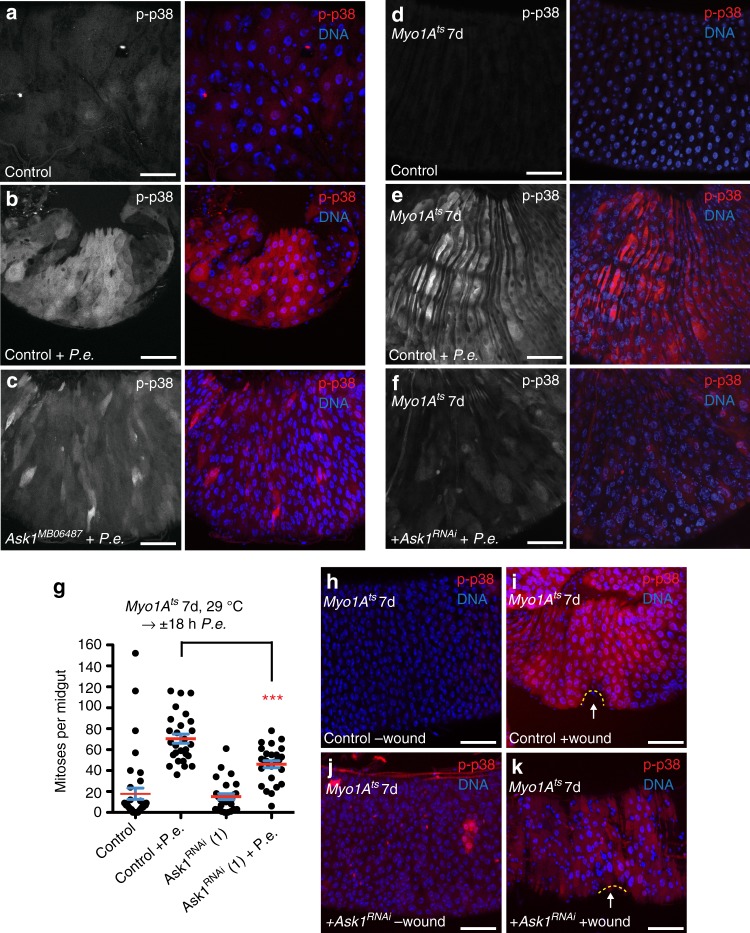
Ask1-p38 signaling in ECs promotes ISC proliferation upon damage. **a**–**c** Ask1 is required for p38 activation in ECs upon *P.e*. infection. p38 activation is increased in ECs in *P.e*.-infected control midguts (**b** right, red) compared with uninfected control midguts (**a**; right, red). p38 activation is blocked in infected *Ask1*^*MB06487*^ midguts (**c** right, red) compared with infected control midguts. **d**–**f** Ask1 is required for p38 activation in ECs upon *P.e*. infection. p38 activation is increased in ECs in *P.e*.-infected control (EC-specific, *Myo1A*^*ts*^) midguts (**e** right, red) compared with uninfected control midguts (**d** right, red). p38 activation is blocked, however, in *P.e*.-infected midguts expressing *Ask1*^*RNAi*^ in ECs for 7 days with *Myo1A*^*ts*^ (**f** right, red) compared with *P.e*.-infected control midguts. **g** Ask1 is required in ECs to promote ISC proliferation upon *P.e*. infection. The mean number of phosphorylated histone H3 Ser 10-positive cells per midgut with s.e.m. in control midguts (*Myo1A*^*ts*^) (*n* = 37 midguts), *P.e*.-infected control midguts (*n* = 27 midguts), midguts expressing *Ask1*^*RNAi*^ in ECs for 7 days with *Myo1A*^*ts*^ (*n* = 27 midguts) and in *P.e.*infected midguts expressing *Ask1*^*RNAi*^ in ECs for 7 days (*n* = 27 midguts) (Mann–Whitney; *p* = 0.0003). Midguts pooled from three independent experiments. **h**–**k** Ask1 is required for p38 activation in ECs upon wounding. p38 is activated in ECs of wounded control midguts (**i** red) compared with unwounded control midguts (**h** red) or unwounded midguts expressing *Ask1*^*RNAi*^ in ECs for 7 days with *Myo1A*^*ts*^ (**j** red). p38 activation is blocked in ECs of wounded midguts expressing *Ask1*^*RNAi*^ in ECs for 7 days with *Myo1A*^*ts*^ (**k** red) relative to ECs in wounded control midguts. DNA is in blue. Scale bars in **a**–**f**, **h**–**k** are 50 μm. Representative images in **a**–**c** from two experiments; **d**–**f**, three experiments; **h**–**k**, two experiments. Source data are provided as a Source Data File

Both JNK and p38 are known to be activated upon stress via upstream SAP3Ks (i.e., Mekk1, Ask1, and Tak1) and SAP2Ks (i.e., MKK4/7 and MKK3/6)^13^. Previously it was reported that p38 was activated in fly ECs via Mekk1 and MKK3 after feeding flies soluble microbial extract^21^. Surprisingly, neither p38 activation nor the ISC response to infection was inhibited upon *P.e*. infection in *Mekk1* mutant midguts (Supplementary Fig. 6a–e) or in midguts depleted for *Mekk1* by *RNAi* in ECs with *Myo1A*^*ts*^ (Supplementary Fig. 6f–m). Since Tak1 signaling is important for AMP production after pathogenic infection^31^, we tested if Tak1 was required for p38 activation in ECs upon infection. We found that neither p38 activation nor the ISC response upon infection are affected in *Tak1* mutant (*Tak1*^*2*^) midguts (Supplementary Fig. 7a–e). However, basal p38 levels in *Tak1* mutant midguts were detectably higher relative to unstressed controls (Supplementary Fig. 7a, c). Since ROS production via Duox has been proposed as a defense to bacterial infection, we tested whether Ask1, a SAP3K thought to sense ROS^32^, was required for p38 activation in ECs. Ask1 was previously shown in *C. elegans* to be required for resistance to pathogenic infection^20^; however, how Ask1 promotes resistance and its requirement in gut epithelial regeneration is unknown. Basal levels of active p38 were not measurably affected in unstressed *Ask1* mutant (*Ask1*^*MB06487*^) midguts, or midguts depleted for *Ask1* in ECs, or expressing an EC-targeted catalytically dead form of Ask1 (Ask1^K618M^)^42^ (Figs. 4a, d, 5a, d, h, j; Supplementary Fig. 8a–d, f, h). However, we found that these treatments all strongly suppressed p38 activation during *P.e*. infection (Fig. 5b, c, e, f; Supplementary Fig. 8e, g, i). Ask1 was also required for p38 activation in ECs after 1% H_2_O_2_ or 0.2% SDS exposure relative to stressed control midguts (Fig. 4b, c, e, f). Next, we determined whether Ask1 was also required in ECs for the ISC response to damage. While *Ask1* depletion alone did not detectably affect basal ISC divisions, *Ask1* depletion in ECs impaired the ISC proliferation response to *P.e*. infection (Fig. 5g). Furthermore, we found that *Ask1* depletion in ECs suppressed p38 activation in ECs, but not the VM, upon wounding (Fig. 5h–k; Supplementary Fig. 9c–j). These data indicate that Ask1 is required for p38 activation upon chemical stress, mechanical stress, and pathogenic bacterial infection. Furthermore, they suggest that Ask1-p38 signaling but not Mekk1- or Tak1-p38 signaling is required in ECs for the ISC response to damage.

### Nox-derived ROS activate p38 and promote midgut regeneration

**Fig. 6:**
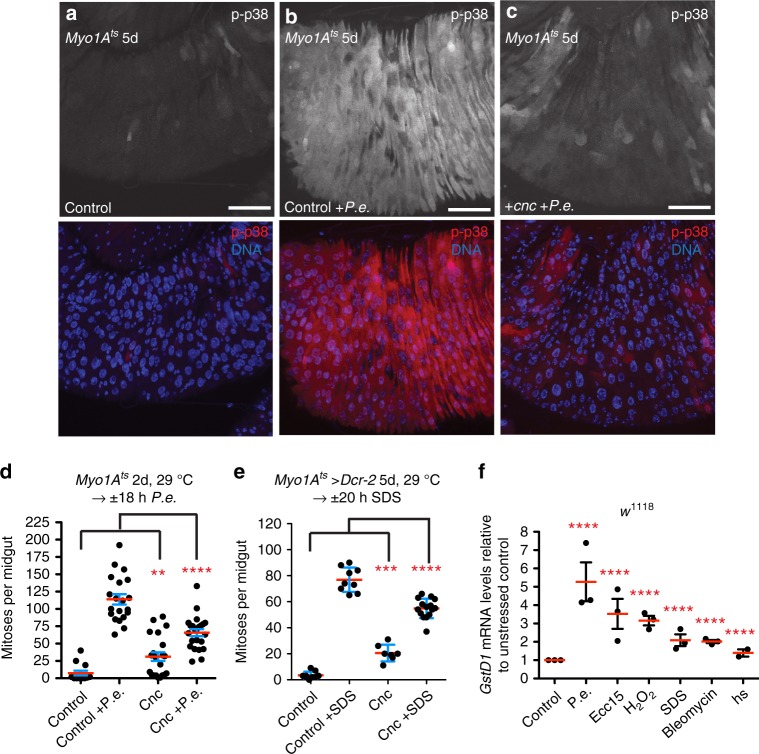
ROS-p38 signaling in ECs promotes ISC proliferation upon damage. **a**–**c** ROS are required for p38 activation in ECs upon *P.e*. infection. p38 activation increases in ECs in *P.e*.-infected control midguts (EC-specific, *Myo1A*^*ts*^) (**b** below, red) compared with uninfected control midguts (**a** below, red). p38 activation is blocked, however, in *P.e*.-infected midguts overexpressing Nrf2 (Cnc) in ECs for 5 days with *Myo1A*^*ts*^ (**c** below, red) compared with *P.e*.-infected control midguts. **d**, **e** Nrf2 overexpression in ECs blocks ISC-mediated regeneration upon *P.e*. infection and detergent exposure. The ISC response is blocked in *P.e*.-infected midguts overexpressing Nrf2 in ECs for 2 days with *Myo1A*^*ts*^ compared with *P.e*.-infected control midguts (Mann–Whitney; *p* < 0.0001). ISC mitoses are increased in midguts overexpressing Nrf2 for 2 days compared with control midguts (Mann–Whitney; *p* = 0.0011). The mean number of phosphorylated histone H3 Ser 10-positive cells per midgut with s.e.m. in control midguts (*Myo1A*^*ts*^) (*n* = 13 midguts), *P.e*.-infected control midguts (*n* = 20 midguts), midguts expressing Nrf2 (*cnc*) in ECs for 2 days with *Myo1A*^*ts*^ (*n* = 22 midguts) and in *P.e.-*infected midguts expressing *cnc* in ECs for 2 days (*n* = 23 midguts). Midguts pooled from two independent experiments. **d** ISC-mediated regeneration is blocked in SDS-fed (20 h) midguts overexpressing Nrf2 (Cnc) in ECs for 6 days with *Myo1A*^*ts*^ compared with SDS-fed control midguts (unpaired *t*; *p* < 0.0001). ISC mitoses are increased in midguts overexpressing Nrf2 for 6 days compared with control midguts (unpaired *t*; *p* < 0.0001). The mean number of phosphorylated histone H3 Ser 10-positive cells per midgut with s.e.m. from one representative experiment in control midguts (*Myo1A*^*ts*^ *>* *Dcr-2*) (*n* = 9 midguts), SDS-fed control midguts (*n* = 9 midguts), midguts expressing Nrf2 (*Cnc*) in ECs for 6 days with *Myo1A*^*ts*^ (*n* = 7 midguts) and in SDS-fed midguts expressing *Cnc* in ECs for 6 days (*n* = 15 midguts) (**e**). **f** Multiple stresses induce ROS-Nrf2 target gene *GstD1* expression. Mean relative *GstD1* mRNA levels with s.e.m. from *n* = 3 independent experiments (one-way ANOVA: *p* = 0.0086; Dunnett’s: *P.e.* < 0.0001, *Ecc15* < 0.0001, H_2_O_2_ < 0.0001, SDS < 0.0001, bleomycin < 0.0001, heat shock < 0.0001) determined by qPCR from stressed and unstressed *w*^*1118*^ midguts. DNA is in blue. Scale bars in **a**–**c** are 50 μm. Representative images in **a**–**c** from three experiments. Source data are provided as a Source Data File

**Fig. 7:**
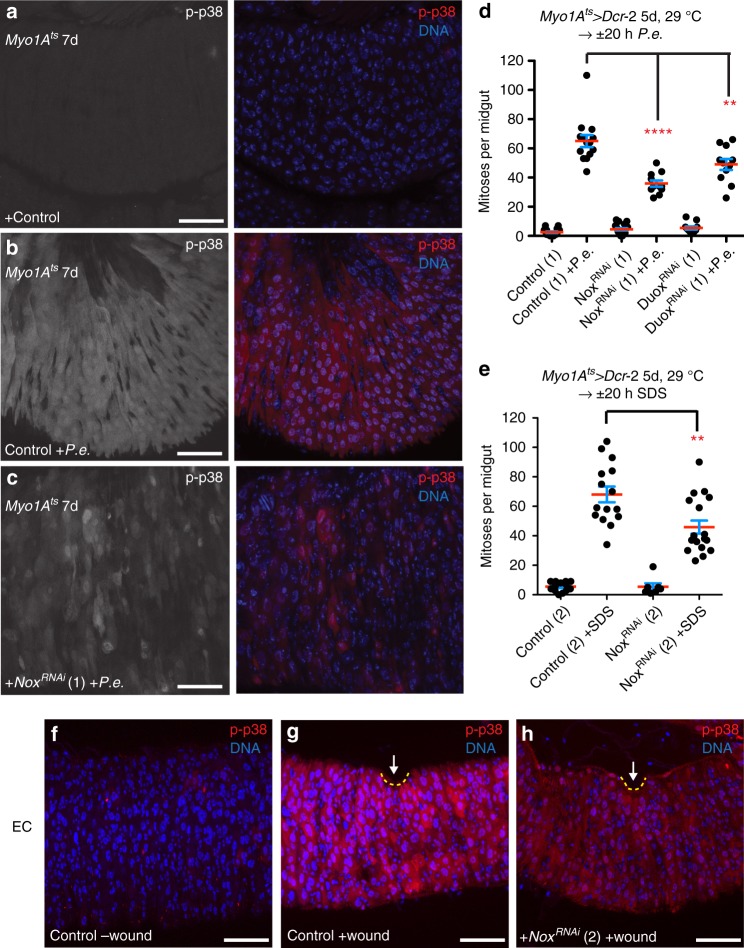
Nox-derived ROS promotes p38 activation and intestinal regeneration. **a**–**c** Nox is required for p38 activation in ECs upon *P.e*. infection. p38 activation increased in ECs of *P.e*.-infected control (*Myo1A*^*ts*^) midguts (**b** right, red) compared with uninfected control midguts (**a** right, red). p38 activation is blocked, however, in *P.e*.-infected midguts expressing *Nox*^*RNAi*^ in ECs for 7 days with *Myo1A*^*ts*^ (**c** right, red) compared with *P.e*.-infected control midguts. **d** Nox and Duox are both required for ISC-mediated regeneration upon *P.e*. infection. The ISC response is blocked in *P.e*.-infected midguts expressing *Nox*^*RNAi*^ (1) or *Duox*^*RNAi*^ (1) for 5 days with *Myo1A*^*ts*^ *>* *Dcr-2* compared with *P.e*.-infected control midguts (unpaired *t*; *p* < 0.0001 (*Nox*^*RNAi*^), *p* < 0.0103 (*Duox*^*RNAi*^). The mean number of phosphorylated histone H3 Ser 10-positive cells per midgut with s.e.m. from one representative experiment in control (1) midguts (*n* = 18 midguts) (*Myo1A*^*ts*^ *>* *Dcr-2* alone), *P.e*.-infected control (1) midguts (*n* = 14 midguts), midguts expressing *Nox*^*RNAi*^ (1) in ECs for 5 days with *Myo1A*^*ts*^ *>* *Dcr-2* (*n* = 19 midguts), *P.e*.infected midguts expressing *Nox*^*RNAi*^ (1) in ECs for 5 days (*n* = 11 midguts), midguts expressing *Duox*^*RNAi*^ (1) in ECs for 5 days with *Myo1A*^*ts*^ *>* *Dcr-2* (*n* = 11 midguts) and *P.e*.-infected midguts expressing *Duox*^*RNAi*^ (1) in ECs for 5 days with *Myo1A*^*ts*^ *>* *Dcr-2* (*n* = 11 midguts). **e** Nox is required in ECs for ISC-mediated regeneration upon detergent exposure. The ISC response is blocked in SDS-fed (20 h) midguts expressing *Nox*^*RNAi*^ (2) for 5 days with *Myo1A*^*ts*^ *>* *Dcr-2* compared with SDS-fed control midguts (unpaired *t* test; *p* < 0.0001). The mean number of phosphorylated histone H3 Ser 10-positive cells per midgut with s.e.m. in control (2) midguts (*n* = 17 midguts from one representative experiment) (*Myo1A*^*ts*^ *>* *Dcr-2* alone), SDS-fed control (2) midguts (*n* = 29 midguts from two experiments), midguts expressing *Nox*^*RNAi*^ (2) in ECs for 5 days with *Myo1A*^*ts*^ *>* *Dcr-2* (*n* = 6 midguts from one representative experiment), and in SDS-fed midguts expressing *Nox*^*RNAi*^ (2) in ECs for 5 days (*n* = 32 midguts from two experiments). **f**–**h** Nox-derived ROS are required for p38 activation in ECs upon wounding. p38 is activated in ECs of wounded control midguts (**g** red) compared with unwounded control midguts (**f** red). p38 activation is blocked in ECs of wounded midguts expressing *Nox*^*RNAi*^ (2) (**h** red) relative to ECs in wounded control midguts. DNA is in blue. Scale bars in **a**–**c**, **f**–**h** are 50 μm. Representative images in **a**–**c** from three experiments; **f**–**h**, two experiments. Source data are provided as a Source Data File

Since ingested ROS could activate Ask1-p38 in midgut ECs, we next tested whether ROS were produced in response to different stresses. Using qPCR, we found that the Nrf2 target, *GstD1*, was induced upon a variety of stresses, suggesting an increase in ROS levels in stressed midguts relative to unstressed midguts (Fig. 6f). Since gut-produced ROS contributes to innate immunity and ISC proliferation in the fly midgut^10,26^, we next tested whether ROS were required for p38 activation after pathogenic infection. To decrease ROS in ECs, we overexpressed Nrf2 (Cnc), a transcription factor that promotes antioxidant factor expression^43^. As an alternative, we co-overexpressed the antioxidant enzymes superoxide dismutase 1 (Sod1), which converts superoxide (O_2_•) to H_2_O_2_, and Catalase, which converts H_2_O_2_ to H_2_O and O_2_. While overexpression of these factors alone moderately increased active p38 levels and ISC proliferation without infection (Fig. 6a, d, e; Supplementary Fig. 10a, b, d), overexpression of these factors inhibited p38 activation during *P.e*. infection (Fig. 6b, c; Supplementary Fig. 10c). Furthermore, the ISC response to *P.e*. infection and detergent exposure was strongly suppressed relative to control by Nrf2 overexpression (Fig. 6d, e), suggesting that ROS are required in ECs to promote ISC-mediated regeneration in response to a variety of damage and not only to pathogens. To identify the molecular source of ROS production, we depleted Nox or Duox, which produce O_2_• and H_2_O_2_, respectively, and contribute to mucosal immunity through ROS generation^22,26,44^. Depletion of *Nox* or *Duox* in ECs of unchallenged midguts neither detectably affected basal active p38 levels nor the basal rate of ISC division (Fig. 7a, d, e; Supplementary Fig. 11a, b, d, f). However, *Nox* depletion was able to inhibit p38 activation during *P.e*. infection relative to infected controls (Fig. 7b, c). *Duox* depletion in ECs did not detectably affect p38 activation upon *P.e*. infection (Supplementary Fig. 11c, e). These data suggest that p38 is activated by Nox-derived ROS produced during pathogenic infection. We then determined if ROS generated during pathogenic infection are required in ECs for ISC proliferation. We found that depletion of either Nox or Duox in ECs partially suppressed the normal proliferative response in ISCs relative to infected control midguts (Fig. 7d; Supplementary Fig. 11f). These data suggest that in addition to promoting pathogen clearance, ROS produced by both Nox and Duox in ECs promote intestinal regeneration. In addition, we found that Nox was required in ECs for optimal ISC responses following detergent damage and p38 activation upon wounding (Fig. 7e–h; Supplementary Figs. 11g, 12). Nox-derived ROS appears to promote regeneration in response to a variety of stresses. Interestingly, we found that Duox was not required in ECs for intestinal regeneration following detergent damage (Supplementary Fig. 11g), suggesting that Duox activity may be specific for pathogen clearance. Together these data suggest that Nox can sense diverse types of damage and stress in ECs, and that Nox-derived ROS promote intestinal regeneration by activating Ask1-MKK3-p38 signaling.

## Discussion

**Fig. 8:**
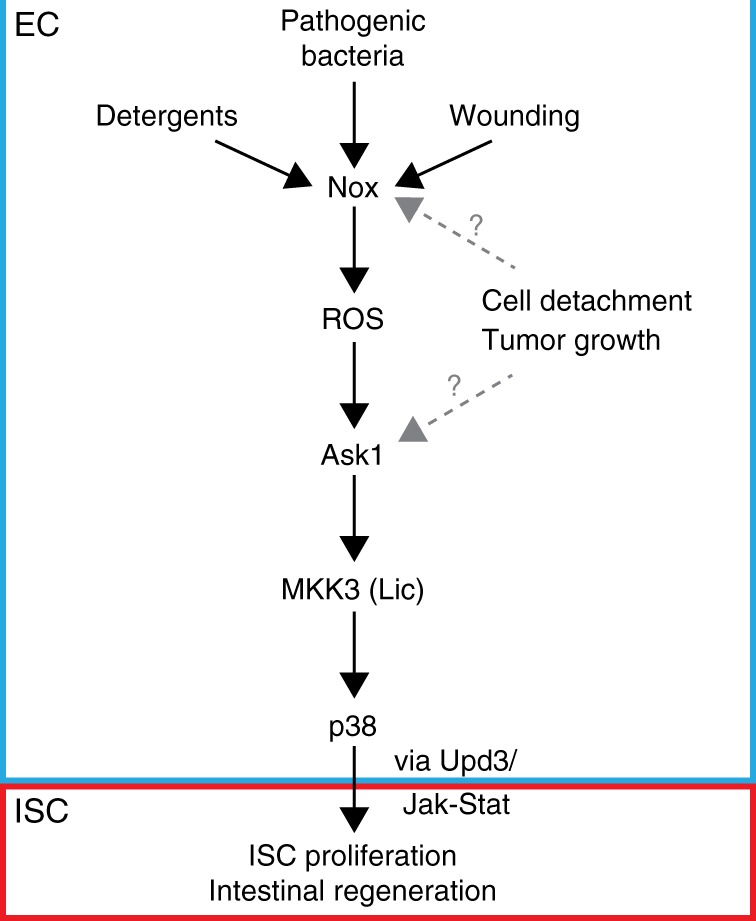
Damage sensing by Nox-Ask1-MKK3-p38 signaling in the fly intestinal epithelial niche. Multiple stresses (pathogenic bacteria, detergents, and wounding) stimulate reactive oxygen species (ROS) production by NADPH oxidase (Nox), thus activating Ask1-MKK3-p38 signaling in enterocytes (EC). Cell detachment and tumor growth may also activate MKK3-p38 signaling either via Nox or Ask1. p38 signaling stimulates Upd3 expression in ECs and JAK-STAT activity in intestinal stem cells (ISCs), thus boosting ISC proliferation and supporting intestinal regeneration

A role for p38 has been reported in intestinal immunity^1^ and in mouse intestinal regeneration upon detergent-induced damage^24,25^. However, how p38 senses stress and promotes intestinal regeneration is not well understood. We found that p38 signaling is activated in ECs during pathogenic bacterial (*P.e*.) infection, oxidative stress (H_2_O_2_), detergent exposure (SDS), wounding, tumor growth, and EC detachment from the BM. In addition, we found that the SAP2K MKK3/Licorne and the SAP3K, Ask1, were required to activate p38 in ECs during *P.e*. infection. Moreover, Ask1 was required for p38 activation in ECs after oxidative stress (H_2_O_2_), detergent (SDS) exposure, and wounding, suggesting that Ask1 may act as a node that integrates multiple types of stress inputs for p38 activation. Using Nrf2 and antioxidant (Sod1 and Catalase) overexpression, we also found that ROS are required for p38 activation during *P.e*. infection. Furthermore, we determined that ROS contributed by the NADPH oxidase, Nox, were required for full p38 activation upon *P.e*. infection and wounding. Surprisingly, Duox, previously also suggested to produce ROS to promote intestinal immunity and repair^10,22^, was not detectably required for p38 activation. Furthermore, Nox, Ask1, and p38 were all required in ECs to stimulate ISC proliferation and regeneration following *P.e*. infection. This stem cell response appeared to be mediated at least in part by Upd3/Jak-Stat signaling. Lastly, Nox-derived ROS was also required for ISC-mediated regeneration upon detergent exposure and p38 activation upon wounding, suggesting that Nox, like Ask1, may also sense multiple different types of epithelial stresses to promote regeneration. Our work suggests that in addition to JNK and Hpo signaling, Nox-Ask1-MKK3-p38 signaling promotes intestinal regeneration in response to multiple different types of stress, thereby enhancing tissue and organismal resilience (Fig. 8). Moreover, since NADPH oxidases (Nox, Duox) are expressed in a variety of cell types, including macrophages^45^, and since Ask1-p38 signaling can potentially be activated in any cell type, our work suggests that Nox-Ask1-MKK3-p38 signaling may promote regeneration in a variety of contexts.

Previous work in the fly midgut reported that Mekk1-p38a-Atf2 and Mekk1-p38c-Atf2 activity promote intestinal immunity by inducing Duox-mediated ROS production^22,23^, and that ROS are required for intestinal regeneration^10^. However, we found that *Mekk1*, *p38a*, *p38c,* and *Atf2* are not required for intestinal regeneration. Instead, we did find that both *p38a* *+* *b* are required for intestinal regeneration, partly by regulating *upd3* expression. Interestingly, *Duox* was also required upon *P.e*. infection for robust intestinal regeneration, suggesting that additional mechanisms may increase Duox activity and that its increased activity upon infection is not solely through its transcriptional upregulation via Atf2. Previous work showed that the survival of infected *p38a* or *p38b* mutant flies upon *P.e*. infection was comparable with control infected flies^23^. We similarly found that the ISC response to infection in *p38a* and *p38c* double-mutant (*p38a*^*1*^) midguts or those depleted for *p38a* in ECs were similar to control infected midguts. These data suggest that neither p38a nor p38c alone are required for intestinal regeneration. Instead, the combination of *p38a* and *p38b* is required in ECs to promote ISC-mediated regeneration. While *p38b* mutant midguts did show a mild defect in the ISC response to infection, loss of *p38b* alone may not reduce organismal resilience to infection. Previous work suggests that p38b partially regulates *upd3* expression^19^. Consistent with this, we found that *upd3* induction is blocked upon infection in midguts expressing *p38b*^*RNAi*^ or *p38a* *+* *b*^RNAi^ in ECs, and that JAK-STAT signaling is blocked in midguts expressing *p38a* *+* *b*^*RNAi*^ in ECs. Together these data suggest that p38a + b signaling promotes intestinal regeneration upon infection via Upd3/JAK-STAT signaling, and not through Atf2-mediated *Duox* expression.

Like p38, JNK in ECs has been shown to sense stress, i.e., pathogenic bacteria, ROS, tumor growth, and EC detachment from the BM. However, unlike p38, JNK can sense heat stress, and heat stress can induce *upd3* expression. While JNK activation in ECs had previously been shown to be sufficient to promote cytokine *upd2-3* expression and ISC proliferation^8^, whether JNK is required in ECs for intestinal regeneration had, surprisingly, not been directly demonstrated. We found that JNK signaling is indeed required in ECs for normal ISC-mediated regeneration upon *P.e*. infection (Supplementary Fig. 13a). In addition to JNK, Hpo signaling is inhibited in response to a variety of stresses and required for intestinal regeneration^7,11,12^. Since depleting Nox, Ask1, or p38 in ECs only partially suppressed regeneration, it seems likely that Nox-Ask1-p38 signaling works together with JNK and/or Hpo/Yki signaling to promote mitogen production and ISC activation. Alternatively, p38 might act via Yki, activating it in ECs by antagonizing Hpo/Wts signaling^46^. To test whether p38 and JNK work together, we depleted *p38a* *+* *b* with *RNAi* and overexpressed the JNK inhibitor, puckered in ECs. We found that blocking p38a + b and JNK with these treatments did not inhibit the ISC response to *P.e*. infection relative to infected controls. Furthermore, we found increased basal ISC divisions without infection in midguts with ECs with reduced p38a + b and JNK activity, confounding our interpretation of this experiment. The details of how these stress-sensing pathways (Jnk, p38, and Hpo) cross-talk and integrate stress responses in the midgut remain an interesting area for future studies.

We found that many different types of stress can activate p38 signaling in adult fly midgut ECs, i.e., Gram-negative pathogenic bacteria (*P.e*.), ROS (H_2_O_2_), detergent (SDS), wounding, ISC tumor growth and EC detachment. Furthermore, we found that Gram-negative pathogenic bacteria, ROS, detergents, and wounding all require Ask1 to activate p38 in ECs. These data suggest that a variety of stresses can be sensed in ECs by Ask1. How Ask1 senses these stresses is not well understood. Ask1 could possibly sense each stress by distinct mechanisms. On the other hand, each stress might also trigger ROS production, possibly via Nox, and thereby activate Ask1. Consistent with this mechanism, we found that a ROS-responsive Nrf2 target, *GstD1*, is induced after infection, detergent, oxidative, DNA damage, and heat shock stress, suggesting that ROS are produced by all these stresses. Furthermore, Nox-derived ROS were required in ECs for p38 activation upon infection and wounding, and for the ISC response to infection and detergent exposure. Thus our data are consistent with the possibility that Ask1 senses damage through Nox-derived ROS to effect intestinal regeneration. Ask1 proteins have been proposed to oligomerize using their C-terminal coiled–coiled domains^47^. Ask1 activity is thought to be regulated by ROS at its N-terminal coil–coil domain, which binds to the redox protein thioredoxin^32^. Thioredoxin binding to the N-terminal coil–coil domain of Ask1 is thought to prevent autophosphorylation and activation of oligomerized Ask1^47^. Upon oxidative stress, ROS oxidize thioredoxin, causing thioredoxin to dissociate from Ask1 and resulting in the activation of oligomerized Ask1 proteins^47^. For this reason, an N-terminally deleted Ask1 (ΔN Ask1) is thought to be constitutively active^32^. An alternate model proposes that Ask1 oxidation results in oligomerization via intermolecular cysteine disulfide bond formation and activation through phosphorylation^48^. In addition, oligomerization has been proposed to be dependent on multiple cysteines throughout Ask1, and interestingly, ΔN Ask1 was able to oligomerize and activate JNK^48^. Furthermore, disulfide bond formation between thioredoxin and Ask1 is thought to result in rapid reduction of Ask1 oligomers to an active monomeric form^48^. However, we found that overexpression of ΔN Ask1^49^ in ECs did not act as a constitutively active form of Ask1 and was unable to strongly activate p38, raising questions about these proposed mechanisms. Surprisingly, we were able to suppress p38 activation during *P.e*. infection by expressing the ΔN Ask1. While we show that Ask1 plays an important role in stress-triggered intestinal regeneration, precisely how these stresses activate Ask1 remains to be determined.

ROS produced by the NADPH oxidase, Duox, in ECs have been reported to promote not only intestinal immunity toward pathogenic bacteria but also intestinal regeneration^10,22^. Similarly, another NADPH oxidase, Nox, also in ECs, has been reported to produce ROS upon exposure to specific commensal bacteria, which subsequently also stimulates ISC proliferation^26^. Nevertheless, whether Nox-derived ROS provides immunity toward either commensal or pathogenic bacteria or whether it normally promotes intestinal turnover remained to be determined. We found that Nox was required in ECs even more than Duox to activate p38 and promote ISC proliferation in response to pathogenic bacteria. This is consistent with our finding that *Nox*, but not *Duox*, mRNA is strongly upregulated (18 × ) in ECs after *P.e*. infection^50^. Our data suggest that Nox plays an important role, possibly as important as Duox, in intestinal immunity and regeneration during pathogenic bacterial infection. It is thought that ROS can directly activate ISC proliferation upon stress^51^. However, our work suggests that a significant contributor to ISC-mediated regeneration is nonautonomous activation of JAK-STAT signaling in ISCs by Nox-ROS-p38 signaling in ECs^52^. Furthermore, our data suggest that unlike Duox, Nox plays an important role in intestinal regeneration following diverse types of damage. An important question remaining is how ROS production by Nox is activated by each stress. Nox could be stimulated by a single cue, such as actin^53^ or by different cues activated by each stress.

Inflammatory bowel diseases (e.g., ulcerative colitis, Crohn’s disease) are characterized by chronic gastrointestinal inflammation due to damage, and have been linked to an increased risk for colorectal cancer. The cause of IBDs and how they develop is unfortunately not well understood. Work in the mouse intestine indicates that p38α signaling is required for intestinal repair after detergent (DSS)-induced damage^24,25^. Furthermore, loss of p38α in the intestinal epithelium enhances carcinogen/DSS damage-induced intestinal tumor initiation^24,54^. Together with our work, these data indicate the importance of the damage sensing and pro-regenerative roles of p38 signaling in intestinal epithelial homeostasis. Thus, a better understanding of how damage is sensed by epithelia and coupled to tissue repair will hopefully yield insights important for the treatment of inflammatory diseases and cancer prevention.

## Methods

### Fly stocks

All experiments were performed using 5–10 -day old, adult, mated female *Drosophila melanogaster* unless stated otherwise. The following fly stocks were used: *w*^*1118*^*, yw, puc-lacZ, upd3-lacZ, 10x STAT-GFP*, *esgGAL4; tubGAL80*^*ts*^ *UAS-GFP* (*esg*^*ts*^)*, esgGAL4 tubGAL80*^*ts*^ *UAS-GFP; UAS-FLP, act* *<* *CD2* *>* *GAL4 (esg*^*ts*^*F/O), Myo1AGAL4; tubGAL80*^*ts*^ *UAS-GFP* (*Myo1A*^*ts*^)*, tubGAL80*^*ts*^ *UAS-GFP; how(24B)GAL4 (how*^*ts*^*), p38a*^*1*^ ^23^ and *p38b*^*ex9*^*; UAS-bsk*^*RNAi*^^55^(34138 GD)*, UAS-Duox*^*RNAi*^ (2)(2593 GD)^23,26^*, UAS-hep*^*RNAi*^ (47507 GD)*, UAS-lic*^*RNAi*^ (2)(106822 KK)*, UAS-Mekk1*^*RNAi*^ (2)(110339 KK)*, UAS-mys*^*RNAi*^ (29619 GD)^9,56^*, UAS-Notch*^*RNAi*^ (27228 GD)^9^, *UAS-Nox*^*RNAi*^ (2)(4914 GD)^26^*, UAS-p38a* *+* *b*^*RNAi*^ ((1) 34238 GD, (2) 52277 GD), *UAS-p38b*^*RNAi*^ (1)(108099 KK) from the Vienna *Drosophila* RNAi Center; *UAS-Ask1*^*RNAi*^ (HMS00464), *UAS-Atf2*^*RNAi*^ (HMC05118, JF02108)*, UAS-Dcr2, UAS-Duox*^*RNAi*^ (1)(HMS00692), *UAS-lic*^*RNAi*^ (1)(HMS05002), UAS-*Mekk1*^*RNAi*^ (1)(HM05075), *UAS-Nox*^*RNAi*^ (1)(HMS00691)*, UAS-p38a*^*RNAi*^ (1)(HMS01224), *Ask1*^*MB06487*^^33^ and *Tak1*^*2*^ ^31^ from the Bloomington Stock Center; *UAS-p38b*^*antisense*^^57^ and *UAS-p38b*^*DN*^^57^ from the Kyoto Stock Center; *UAS-Ask1*^*K618M*^^42^ from Masayuki Miura (University of Tokyo, Japan), *Atf2*^*PB*^^34,41^ from Shunsuke Ishii (RIKEN Tsukuba Institute), *UAS-Cnc*^58^ from Dirk Bohmann (University of Rochester, USA), *UAS-lic* from the Zürich ORFeome project (FlyORF), *MK2*^*Δ1A*^ and *MK2*^*Δ43*^ ^40^ from Hugo Stocker (ETH Zürich, Switzerland), *Mekk1*^*Ur36*^^21,23^ from Shunsuke Ishii (RIKEN Tsukuba Institute), *UAS-Sod1*, *UAS-Catalase*^34^ from Florenci Serras (University of Barcelona, Spain).

### Stress experiments

*P.e*. infection: flies were treated for 18 h with food laced with either 500–650 μl of 5% sucrose or 20 × *P.e*. resuspended in 5% sucrose from an 18 h culture grown at 30 °C, 130 RPM or treated on filter paper with either 450 μl of 5% sucrose or 5% sucrose containing *P.e*. resuspended from a 20–24 h culture grown at 29 °C, 200 RPM. *Erwinia caratova caratova 15* (*Ecc15*) infection: flies were treated on the filter paper with either 450 μl of 5% sucrose or 5% sucrose containing *Ecc15* (OD = 200) resuspended from a 20–24 h culture grown at 29 °C, 200 RPM. Hydrogen peroxide exposure: 1% final of stabilized H_2_O_2_ (Sigma-Aldrich) or H_2_O was mixed into food or fed on filter paper soaked with 450 μl of either 5% sucrose or 0.8% H_2_O_2_ in 5% sucrose. Detergent exposure: 0.2% sodium dodecyl sulfate (SDS) or H_2_O was mixed into food or fed on the filter paper soaked with 450 μl of either 5% sucrose or 0.5% SDS in 5% sucrose. DNA damage: flies were fed on filter paper soaked with 450 μl of either 5% sucrose or 500 μM bleomycin sulfate (Abcam) in 5% sucrose. Heat shock: flies were heat shocked for 30 min or 4 h at 37 °C.

### *Drosophila* genetics

Flies raised at 18 °C were shifted to 29 °C to induce *UAS* transgene expression in progenitors with *esgGAL4; tubGAL80*^*ts*^ *UAS-GFP*, ECs with *Myo1AGAL4; tubGAL80*^*ts*^ (*Myo1A*^*ts*^), and in VM with *how*-GAL4; *tubGAL0*^*ts*^ (*how*^*ts*^). No statistical method was used to predetermine sample size, but typically between 10 and 20 flies were used per experiment. When selecting animals for an experiment, the parental genotype was not concealed since it was required to select relevant progeny. Animals were first selected for their genotype and then randomly chosen to be used for an experiment. See Supplementary Methods for genotypes.

### Histology

After fixation in 8% formaldehyde (+ phosphatase inhibitors: PhosSTOP (Roche)), midguts were washed in PBS, 0.1% Triton X-100, blocked in PBS, 0.1% Trinton X-100, 1% bovine serum albumin (BSA), 2% normal goat serum (NGS), and stained in blocking solution with rabbit polyclonal anti-phospho-p38 (Cell Signaling #9211, 1:50), rabbit polyclonal anti-phospho Ser 10 histone 3 (Upstate Biotechnology/Millipore #06-570, 1:1000), chicken polyclonal anti-β-galactosidase (Abcam #ab9361, 1:1000), and chicken polyclonal anti-GFP (Life Technologies #A10262, 1:1000). Midguts were then washed in PBS 0.1% Triton X-100, stained in PBS, 0.3% Triton X-100, 0.1% BSA with Alexa Fluor-conjugated secondary antibodies (Life Technologies, 1:1000) and Hoechst 33258 (Life Technologies), and mounted in Vectashield (Vector Laboratories).

Mitotic indices were determined by counting the number of pH3-positive cells from whole female midguts from two or more independent experiments. The mean number of mitoses per midgut and s.e.m. are presented for each genotype or treatment. Before quantifying the number of mitoses per midgut, the genotype of each sample was concealed. Samples were then randomly analyzed, and the genotype was revealed only after completing analysis.

### Statistical analysis

Statistical analyses were performed using GraphPad Prism 5 or 8. For data describing mitoses per midgut, the two-sided Mann–Whitney test (for non-Gaussian data) or the two-sided unpaired *t* test (for Gaussian data) was applied to determine statistical significance. For qPCR data, the one-way ANOVA test and the Dunnetts sub-test were used on relative mRNA levels not normalized to control. The one sample *t* test was used on relative mRNA levels normalized to control or treated control. For Stat-GFP or phospho-p38 levels, unpaired *t* test was used on relative levels not normalized to control. The significance level is indicated by an * for *p* ≤ 0.05, ** for *p* ≤ 0.01, *** for *p* ≤ 0.001, **** for *p* ≤ 0.0001, and by NS for not significant, *p* > 0.05.

### Microscopy

Samples were analyzed using Leica M205FA, DM5000B, SP5 and SP8 microscopes. Images were processed with ImageJ (NIH) and Adobe Photoshop CS5. Confocal images are presented as maximal intensity projections of images obtained every 0.5–1.0 μm. When comparing protein levels, each z-stack was acquired with the same laser intensity and gain, maximal intensity projections of identical dimensions were created and were further similarly processed with Adobe Photoshop CS5. Representative images presented were obtained from ≥ 2 independent experiments.

### Image quantitation

Maximal intensity projections (of 5–6 μm thickness) of confocal images from the EC layer of each midgut were generated with ImageJ (NIH). ImageJ was then used to measure the surface area and the signal integrated density for each midgut. The ratio of integrated density:area was determined for each midgut and then normalized to the mean integrated density/area of control midguts to determine the relative signal (phospho-p38 or GFP) levels between treatments and/or genotypes.

### Quantitative RT-PCR

RNA was isolated from 20 midguts using an RNAeasy kit (Qiagen) or from 12 midguts using the RNAquous Kit (Ambion). The concentration and quality of the RNA were determined using the Agilent 2200 Tape Station. In all, 250–500 ng of RNA was used for cDNA synthesis using the Quantitect cDNA synthesis kit (Qiagen) or the SuperScript III kit (Invitrogen). qPCR was performed using the LightCycler 480 SYBER Green I Master (Roche), the Maxima SYBER Green/ROX Master (Thermo Fisher Scientific) or the iTaq Universal SYBR Green Supermix (BioRad). SYBR green incorporation during PCR was detected using the Roche LightCycler 480 II, the Applied Biosystems Step One Plus and the CFX384 Touch Real-Time system (BioRad). The following primers were used: *upd1* F: 5′-CCACGTAAGTTTGCATGTTG-3′, *upd1* R: 5′-CTAAACAGTAGCCAGGACTC-3′, *upd2* F: 5′-CACAAGTGCGGTGAAGCTAA-3′, *upd2* R: 5′-GGCTCTTCTGCTGATCCTTG-3′, *upd3* F: 5′-GCCCTCTTCACCAAACTGAA-3′, *upd3* R: 5′-TTTCTTCTGGATCGCCTTTG-3′ from^9^; *Socs36E* F: 5′-CAGTCAGCAATATGTTGTCG-3′, *Socs36E* R: 5′-ACTTGCAGCATCGTCGCTTC-3′ from^8^ and *Ask1* F: 5′-TTACACTCTCAGCTGCGACA-3′, *Ask1* R: 5′-TGGAGTAGAAGGCCATGGTG-3′, *GstD1* F: 5′- AGAAGTACGGCAAGACCGAC-3′, *GstD1* R: 5′-CTTGAAGGCCTCTGGATCGG-3′, *Mekk1* F: 5′-CGAGTGCTCTCACAGCCATA-3′, *Mekk1* R: 5′-GCCAGTCCTCGACTTTTGAG-3′, *Tak1* F: 5′-CAGGCAGCCTATGTGGACTT-3′, *Tak1* R: 5′-GCTTCACCTCCTTCTCGATG-3′, *lic* F: 5′-ACCGCCTTGTAATGGATTTG-3′, *lic* R: 5′-GCAGATCGTGAAGGAAGAC-3′, *p38a* F:CAGGTGTCAAAGGCAGTTGTT, *p38*a R: 5′-GCTCCCGGTACGTCCTCTT-3′, *p38b* F: 5-TACGACCAGAGCTTCGAGGA-3′ and *p38b* R: 5′-GGCCGGCATTACTGCTCTTT-3′. We found that *crq* and *mk2* gene expression did not change after 18 h *P.e*. infection. In addition, *crq* mRNA levels did not change upon all stresses tested (*Ecc15*, SDS, bleomycin, heat shock, H_2_O_2_, or paraquat). Thus the expression of each target gene was normalized to *crq* or *mk2* expression (reference) to ensure equivalent RNA or cDNA input. The primers used to detect *crq* and *mk2* expression were: *crq* F: 5′-CAGAGCTCTCCTCCGAATTG-3′, *crq* R: 5′-ATGCCGGTGATGAGAAAGAC-3′, *mk2* F: 5′-GGCAAAGGATCTGATCAAGG-3′, and *mk2* R: 5′-GTTTCCTCGGCCTCCTTTAG-3′. Each assay was performed in triplicate or quadruplet on ≥ 2 independent biological replicates. All data are presented as relative mRNA levels compared with control (translated from raw Ct values), fold change = 2^−∆∆Ct^ or % decrease relative to control with mean and s.e.m.

### Reporting summary

Further information on research design is available in the Nature Research Reporting Summary linked to this article.

## Supplementary information

Supplementary Information

Reporting Summary

Source Data

## Data Availability

All data are available from the corresponding authors upon request. The source data underlying Figs. 1c–d, g, 2a–c, g, 5g, 6d–f, and 7d–e and Supplementary Figs. 1g–h, j, 2e–i, 3c, e, 4a–c, 6e, l-m, 7e, 8e, 10d, 11f–g, and 13 are provided as a Source Data file.
