# Supplementary material for: Damage sensing by a Nox-Ask1-MKK3-p38 signaling pathway mediates regeneration in the adult Drosophila midgut
Source: Nat Commun. 2019 Sep 25;10:4365. doi: 10.1038/s41467-019-12336-w (PMC6761285; doi:10.1038/s41467-019-12336-w)
Supplement: Supplementary file 1 — Supplementary Information [file 41467_2019_12336_MOESM1_ESM.pdf]

Supplementary Information

Damage sensing by a Nox-Ask1-MKK3-p38 signaling pathway mediates intestinal regeneration in the adult *Drosophila* midgut.

Patel et al.

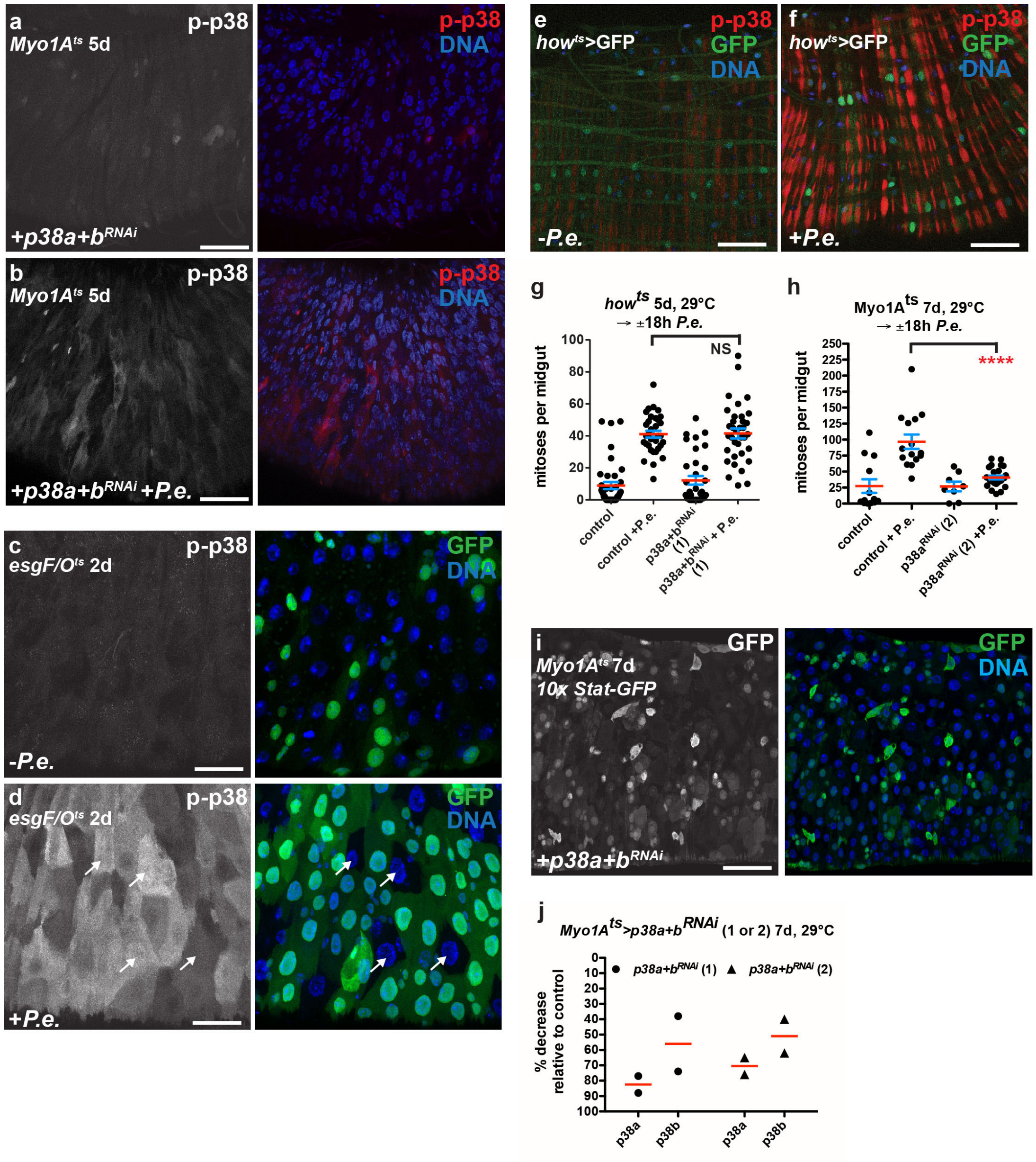

Supplementary Figure 1. p38 activity increases in enterocytes and visceral muscle upon damage

a-b. p38 activation was blocked in *p38a+b* depleted enterocytes upon damage. p38 activity did not increase in uninfected midguts expressing *p38a+b*^{RNAi} in ECs for 5 days with *Myo1A*^{ts} (a; right, red). p38 activation (b; right, red) was blocked in *P.e.* infected midguts expressing *p38a+b*^{RNAi} in ECs for 5 days with *Myo1A*^{ts} compared to *P.e.* infected control midguts (see Figure 1).

c-d. p38 activation (d) increases in both old and new enterocytes (ECs) (d; right, GFP) upon *P.e.* infection compared to control midguts (c). Progenitors (ISCs, EBs) are marked by expressing GFP for 2 days with esg^{ts}F/O (c; right, green): progenitors and new cells (ECs, EEs) are marked by GFP upon *P.e.* infection (d; right, green). Arrows indicate GFP-negative old ECs.

e-f. Increased activated p38 (f, red) is observed in the visceral muscle (VM) upon *P.e.* infection compared to control (e, red). VM marked by expressing GFP (e-f; green) with VM-specific, *how*^{ts}.

g. p38 signaling is not required in the visceral muscle (VM) to promote ISC-mediated regeneration. The ISC response is similar between *P.e.* infected midguts expressing *p38a+b*^{RNAi} in VM for 5 days with *how*^{ts} and *P.e.* infected control midguts (Mann-Whitney; P=0.9853). Mean number of phosphorylated histone H3 Ser 10- positive cells per midgut with s.e.m. in control midguts (VM-specific, *how*^{ts} alone) (n=39 midguts), *P.e.* infected control midguts (n=33 midguts), midguts expressing *p38a+b*^{RNAi} for 5 days with VM-specific *how*^{ts} (n=34 midguts) and in *P.e.* infected midguts expressing *p38a+b*^{RNAi} for 5 days with *how*^{ts} (n=35 midguts). Midguts pooled from 2 independent experiments. NS, not significant.

h. Both p38a and p38b signaling are required in ECs for ISC-mediated regeneration. The ISC response is inhibited in *P.e.* infected midguts expressing *p38a+b*^{RNAi} (2) in ECs for 7 days with *Myo1A*^{ts} relative to *P.e.* infected control midguts (Mann-Whitney; P=<0.0001). Mean number of phosphorylated histone H3 Ser 10- positive cells per midgut with s.e.m. in control midguts (*Myo1A*^{ts}) (n=13 midguts), *P.e.* infected control midguts (n=15 midguts), midguts expressing *p38a+b*^{RNAi} (2) for 7 days (n=8 midguts) and *P.e.* infected midguts expressing *p38a+b*^{RNAi} (2) for 7 days (n=26 midguts). Midguts pooled from 2 independent experiments.

i. Stat activity was not detectably affected in midguts bearing *10X Stat-GFP* and expressing *p38a+b*^{RNAi} in ECs for 7 days with *Myo1A*^{ts} (i; right, green).

j. p38a and p38b mRNA levels are depleted in midguts expressing *p38a+b*^{RNAi} (1 and 3). Mean % decrease relative to control from 2 independent experiments in *p38a* and *p38b* mRNA levels determined by qPCR from midguts expressing *p38a+b*^{RNAi} (1) or (2) in ECs for 7 days with *Myo1A*^{ts}.

DNA is in blue. Scale bars in a-b, e-f, and i are 50 µm; in c-d, 30 µm. Representative images in a-b from 3 experiments; c-d, 2 experiments; e-f, 3 experiments; i, 3 experiments. Source data are provided as a Source Data File.

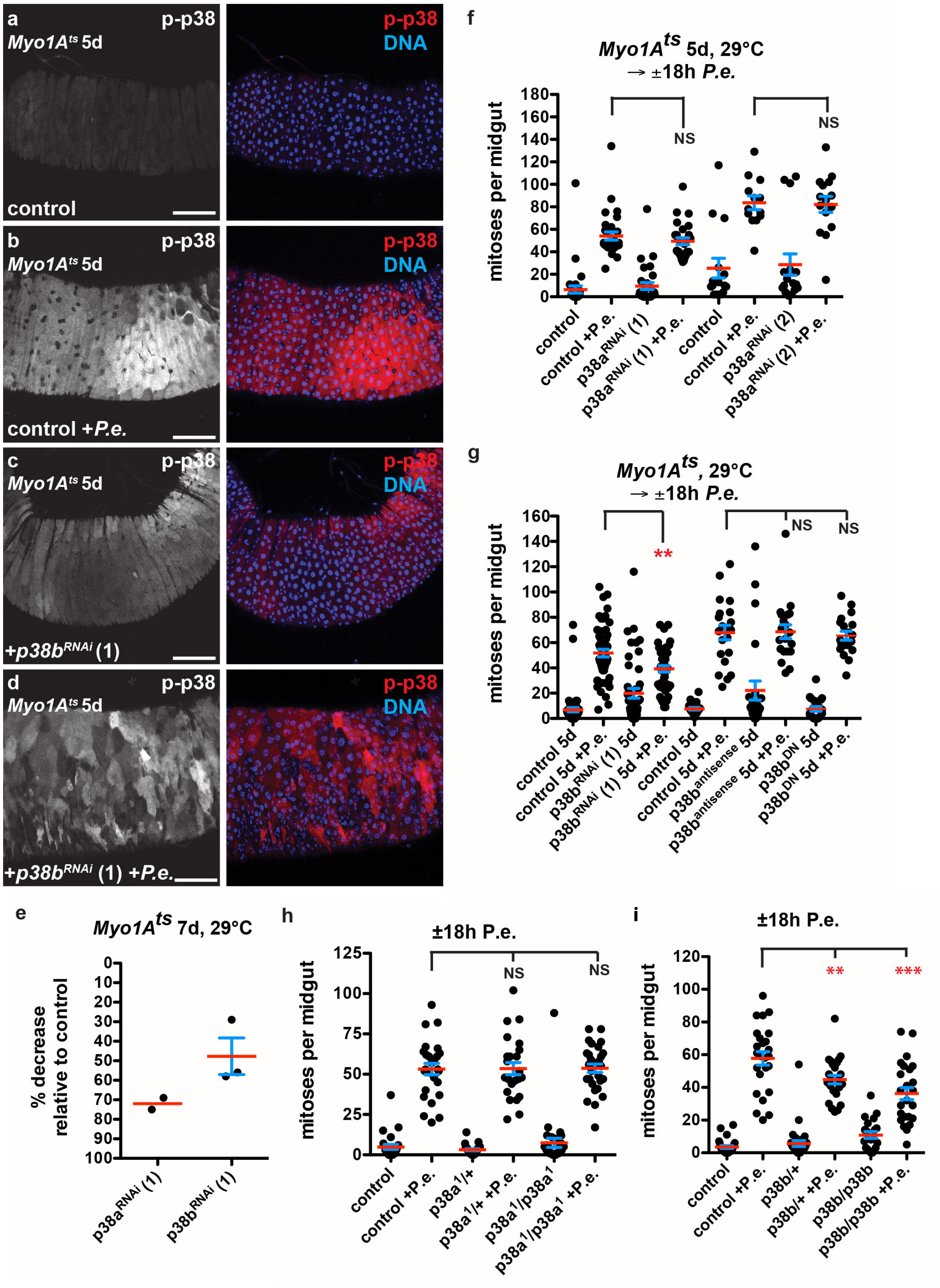

Supplementary Figure 2. p38b, but neither p38a nor p38c, signaling in enterocytes promotes ISC-mediated regeneration

a-d. Depleting p38b in ECs does not affect p38 activation upon damage. p38 activation increased in ECs in *P.e.* infected control (*Myo1A*^{ts}) midguts (b; rightb', red) compared to uninfected control midguts (a; right, red) or uninfected midguts expressing *p38b*^{RNAi} in ECs for 5 days with *Myo1A*^{ts} (c; right, red). Increased p38 activation was also found in *P.e.* infected midguts expressing *p38b*^{RNAi} in ECs for 5 days with *Myo1A*^{ts} (d; right, red) similar to *P.e.* infected control midguts.

e. *p38a* and *p38b* mRNA levels are depleted in midguts expressing *p38a*^{RNAi} (1) and *p38b*^{RNAi} (1) in ECs. Mean % decrease relative to control from 2 (*p38a*^{RNAi}) or with s.e.m from 3 (*p38b*^{RNAi}) independent experiments in *p38a* or *p38b* mRNA levels determined by qPCR from midguts expressing *p38a*^{RNAi} (1) and *p38b*^{RNAi} (1) in ECs for 7 days with *Myo1A*^{ts}.

f. Blocking p38a signaling in ECs does not affect ISC-mediated regeneration. ISC proliferation was similar between *P.e.* infected midguts expressing *p38a*^{RNAi} (1 and 2) in ECs for 5 days with *Myo1A*^{ts} and *P.e.* infected control midguts (Mann-Whitney; P= 0.2663 (1), P=0.8718 (2)). Mean number of phosphorylated histone H3 Ser 10-positive cells per midgut with s.e.m. in control (1 or 2) midguts (*Myo1A*^{ts} alone) (n=32 midguts (1) pooled from 2 independent experiments, n=15 midguts (2)), *P.e.* infected control (1 or 2) midguts (n=30 midguts (1) pooled from 2 independent experiments, n=15 midguts (2)), midguts expressing *p38a*^{RNAi} (1 or 2) in ECs for 5 days with *Myo1A*^{ts} (n=30 midguts (1) pooled from 2 independent experiments, n=16 midguts (2)) and in *P.e.* infected midguts expressing *p38a*^{RNAi} (1 or 2) in ECs for 5 days with *Myo1A*^{ts} (n=27 midguts (1) pooled from 2 independent experiments, n=15 midguts (2)). NS, not significant.

g. Blocking p38b signaling in ECs mildly blocks ISC-mediated regeneration. The ISC response was similar between *P.e.* infected midguts expressing *p38b*^{antisense} or *p38b*^{DN} in ECs for 5 days with *Myo1A*^{ts} and *P.e.* infected control midguts (Mann-Whitney; P=1.0000, P=0.7247), but is mildly blocked in *P.e.* infected *p38b*^{RNAi} expressing midguts compared to *P.e.* infected control midguts (Mann-Whitney; P=0.0044). Mean number of phosphorylated histone H3 Ser 10- positive cells per midgut with s.e.m. in control (1 and 2) midguts (*Myo1A*^{ts}) (n=55 midguts (1), n=22 midguts (2)), *P.e.* infected control midguts (n=53 midguts (1), n=21 midguts (2)), midguts expressing *p38b*^{RNAi} (1) (n=45 midguts) or *p38b*^{antisense} (n=24 midguts) or *p38*^{DN} (n=21 midguts) in ECs for 5 days with *Myo1A*^{ts}, and in *P.e.* infected midguts expressing *p38b*^{RNAi} (1) (n=46 midguts) or *p38b*^{antisense} (n=20 midguts) or p38^{DN} (n=19 midguts) in ECs for 5 days with *Myo1A*^{ts} . Midguts pooled from 2 independent experiments. NS, not significant.

h. *p38a* and *p38c* are not required for ISC-mediated regeneration. The ISC response was similar between *P.e.* infected control, *p38a*$^{1/+}$ and *p38a*$^{1/1}$ midguts (Mann-Whitney; P= (0.7557 (*p38a*$^{1/+}$); P=0.9019 (*p38a*$^{1/1}$)). Mean number of phosphorylated histone H3 Ser 10-positive cells per midgut with s.e.m. in control midguts (n=27 midguts), *P.e.* infected control midguts (n=26 midguts), *p38a*$^{1/+}$ midguts (n=25 midguts), *P.e.* infected *p38a*$^{1/+}$ midguts (n=25 midguts), *p38a*$^{1/1}$ midguts (n=31 midguts) and *P.e.* infected *p38a*$^{1/1}$ midguts (n=30 midguts). Midguts pooled from 3 independent experiments. NS, not significant.

i. *p38b* is required for ISC-mediated regeneration. The ISC reponse was blocked in *P.e.* infected *p38b*$^{ex9/+}$ and *p38b*$^{ex9/ex9}$ midguts (Mann-Whitney; P=0.0090 (*p38b*$^{ex9/+}$); P=0.0007(*p38b*$^{ex9/ex9}$)) compared to infected control midguts. Mean number of phosphorylated histone H3 Ser 10-positive cells per midgut with s.e.m. in control

midguts (n=28 midguts), *P.e.* infected control midguts (n=25 midguts), *p38b*$^{ex9/+}$ midguts (n=31 midguts), *P.e.* infected *p38b*$^{ex9/+}$ midguts (n=25 midguts), *p38b*$^{ex9/ex9}$ midguts (n=21 midguts) and *P.e.* infected *p38b*$^{ex9/ex9}$ midguts (n=25 midguts). Midguts pooled from 3 independent experiments. NS, not significant.

DNA is in blue. Scale bars in a-d are 50 µm. Representative images in a-d from 2 experiments. Source data are provided as a Source Data File.

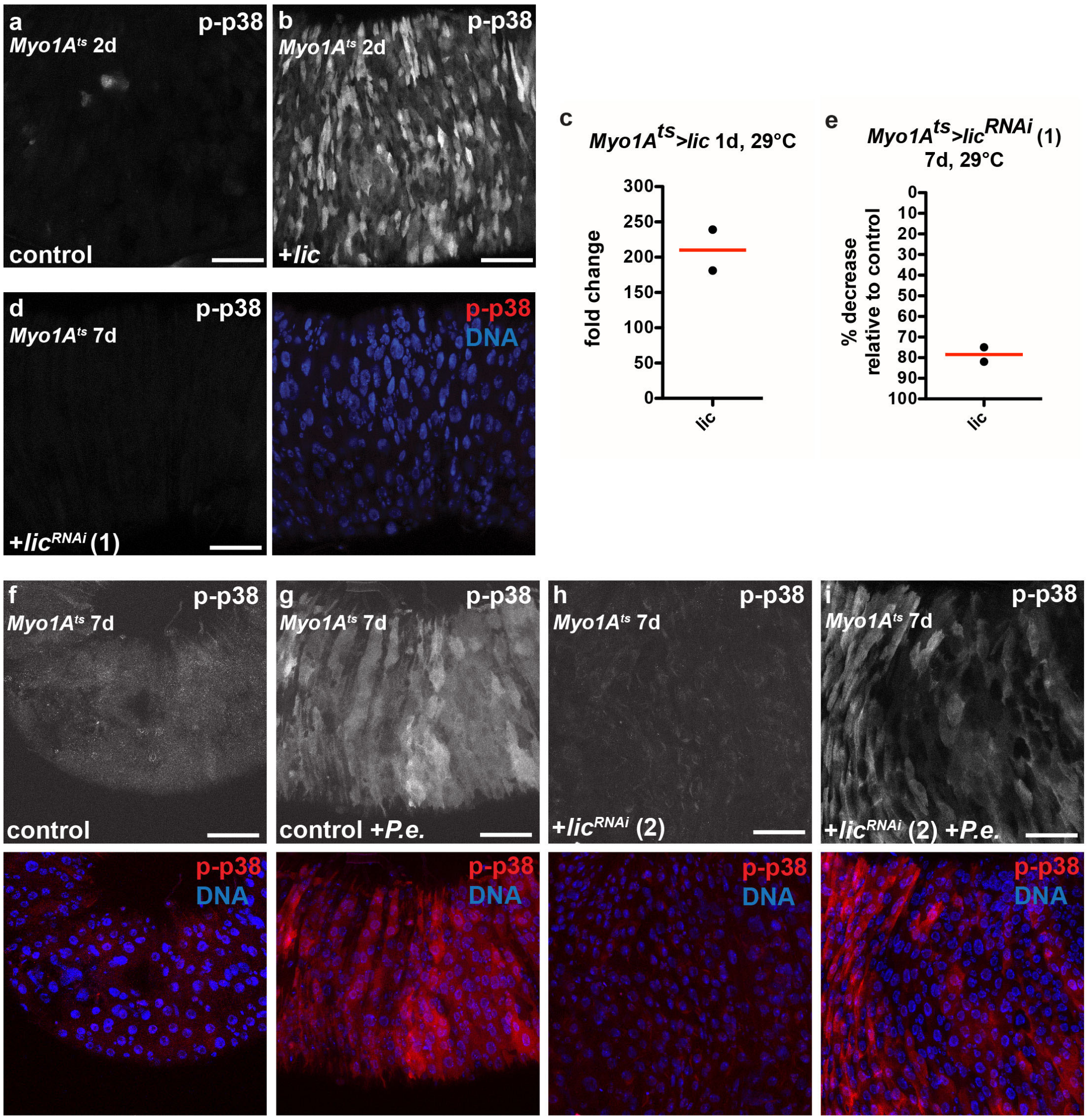

Supplementary Figure 3. MKK3/Licorne is required for p38 activation in ECs upon pathogenic infection

a-b. Mkk3/Licorne can activate p38 in ECs. p38 activation increased in midguts overexpressing Licorne in ECs for 2 days with *Myo1A*^{ts} (b) compared to control midguts (a).

c. *Lic* mRNA levels are increased in midguts overexpressing Lic. Mean fold change from 2 independent experiments in *lic* mRNA levels determined by qPCR from midguts overexpressing *lic* in ECs for 1 day with *Myo1A*^{ts}.

d. p38 activation did not increase in uninfected midguts expressing *lic*^{RNAi} (2) in ECs for 7 days with *Myo1A*^{ts} (d; right, red).

e. *lic* mRNA levels are depleted in midguts expressing *lic*^{RNAi} (1). Mean % decrease relative to control from 2 independent experiments in *lic* mRNA levels determined by qPCR from midguts expressing *lic*^{RNAi} (1) in ECs for 7 days with *Myo1A*^{ts}.

f-i. Mkk3/Licorne is required for p38 activation in ECs upon *P.e.* infection. p38 activation increases in ECs in *P.e.* infected control (*Myo1A*^{ts}) midguts (g; right, red) compared to uninfected control midguts (f; right, red) or midguts expressing *Lic*^{RNAi} (2) in ECs for 7 days (h; right, red). p38 activation (i; right, red) is suppressed however in *P.e.* infected midguts expressing *lic*^{RNAi} (2) for 7 days with *Myo1A*^{ts} compared to *P.e.* infected control midguts.

DNA is in blue. Scale bars in a-e are 50 µm. Representative images in a-b from 3 experiments; d, 2 experiments; f-i, 1 experiment. Source data are provided as a Source Data File.

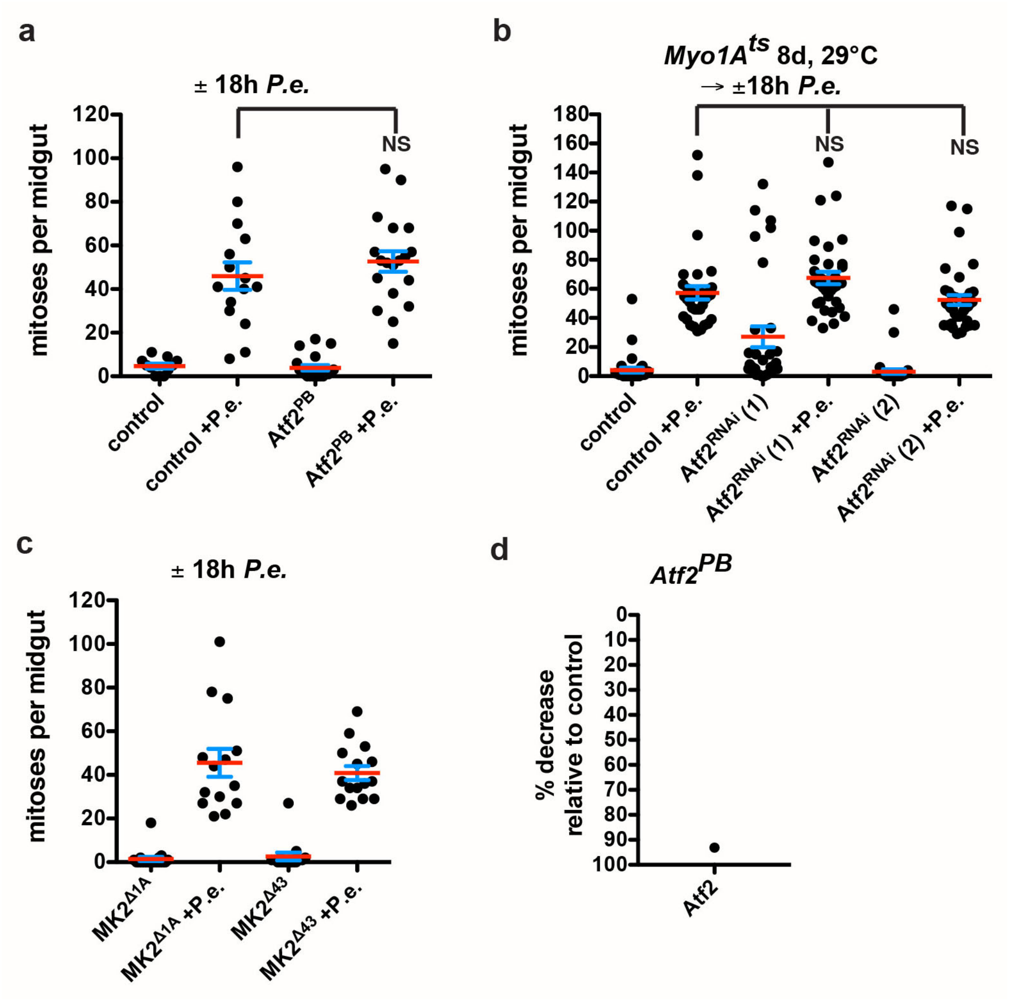

Supplementary Figure 4. Atf2 and MAPK-activated protein kinase 2 (MK2) do not promote ISC-mediated regeneration upon infection

a. Atf2 activity is not required for ISC-mediated regeneration. ISC proliferation was similar between *P.e.* infected $Atf2^{PB/PB}$ midguts and *P.e.* infected control midguts (Mann-Whitney; P=0.3312). Mean number of phosphorylated histone H3 Ser 10-positive cells per midgut with s.e.m. in uninfected control midguts (n=11 midguts), *P.e.* infected control midguts (n=15 midguts), uninfected $Atf2^{PB/PB}$ midguts (n=19 midguts) and *P.e.* infected $Atf2^{PB/PB}$ midguts (n=19 midguts). Midguts pooled from 2 independent experiments. NS, not significant.

b. Atf2 activity is not required in ECs for ISC-mediated regeneration. ISC proliferation was similar between *P.e.* infected midguts expressing $Atf2^{RNAi}$ (1 and 2) for 7 days with $Myo1A^{ts}$ and *P.e.* infected control midguts (Mann-Whitney; P=0.0101 (1), P=0.3100 (2)). Mean number of phosphorylated histone H3 Ser 10- positive cells per midgut with s.e.m. in control midguts ($Myo1A^{ts}$) (n=35 midguts), *P.e.* infected control midguts (n=33 midguts), midguts expressing $Atf2^{RNAi}$ (1 or 2) in ECs for 7 days with $Myo1A^{ts}$ (n=31 midguts (1), n=35 midguts (2)), and *P.e.* infected midguts expressing $Atf2^{RNAi}$ (1 or 2) in ECs for 7 days with $Myo1A^{ts}$ (n=35 midguts (1), n=38 midguts (2)). Midguts pooled from 2 independent experiments. NS, not significant.

c. MK2 signaling is not required for ISC-mediated regeneration. ISC proliferation was similar between *P.e.* infected MK2 mutants (*MK2*$^{\Delta 43}$) and *P.e.* infected control midguts (*MK2*$^{\Delta 1A}$) (Mann-Whitney; P=0.9826). Mean number of phosphorylated histone H3 Ser 10- positive cells per midgut with s.e.m. in control midguts (*MK2*$^{\Delta 1A}$) (n=19 midguts), *P.e.* infected control midguts (n=14 midguts), *MK2* mutant midguts (*MK2*$^{\Delta 43}$) (n=15 midguts) and *P.e.* infected *MK2* mutant midguts (n=15 midguts). Midguts pooled from 2 independent experiments. NS, not significant.

d. *Atf2* mRNA levels were decreased in *Atf2*^{PB} midguts. Mean fold change from 1 experiment in *Atf2* mRNA levels determined by qPCR from *Atf2*^{PB} midguts relative to control.

Source data are provided as a Source Data File.

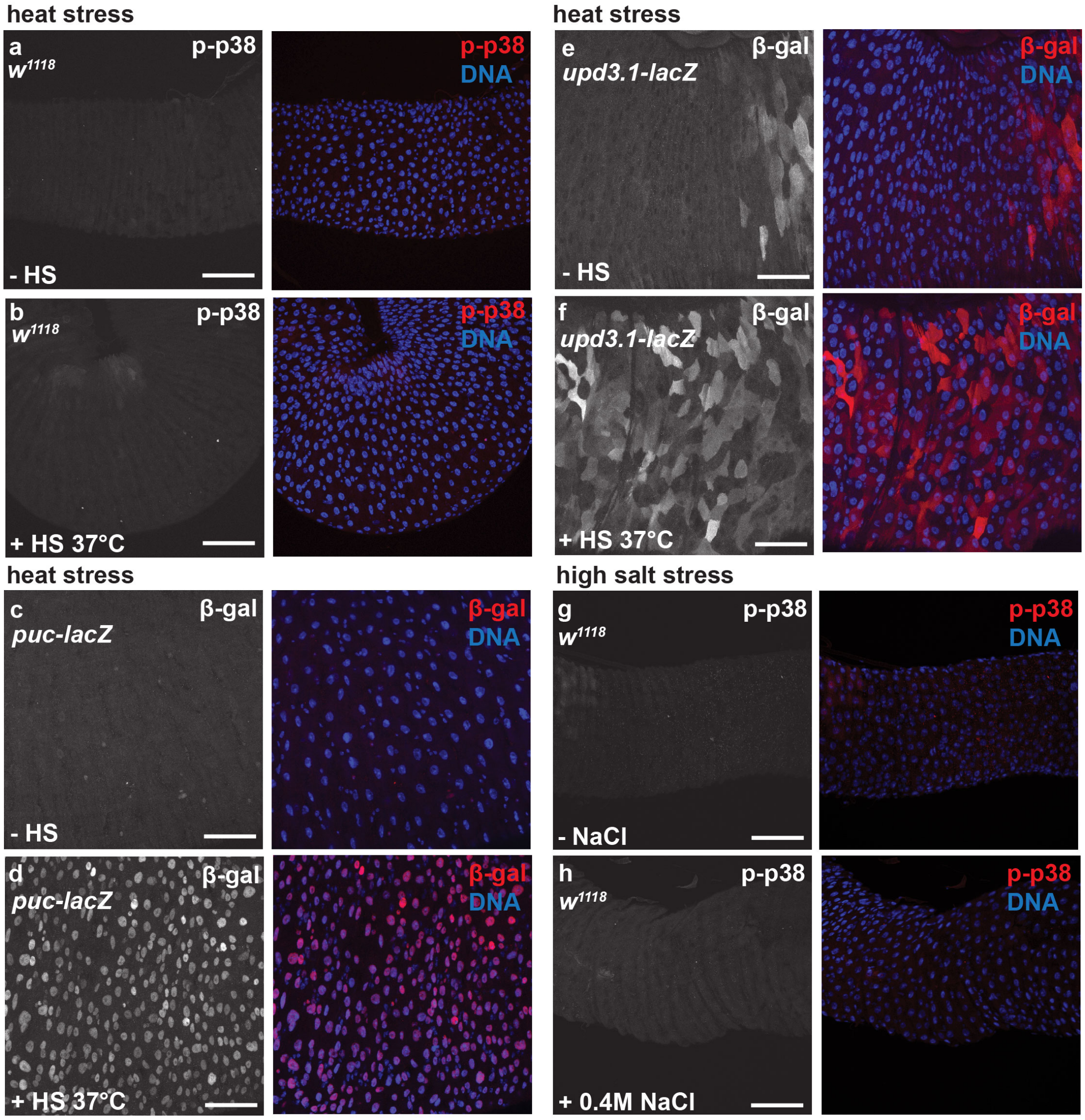

Supplementary Figure 5. Heat and high salt stresses do not activate p38 in ECs

a-f. JNK, but not p38, activity increases in ECs upon heat stress. p38 activation did not detectably increase in ECs in heat shocked (37°C, 30 min) w^{1118} midguts (b; right, red) compared to non-heat shocked w^{1118} midguts (a; right, red). JNK activity (*puckered* expression) was increased in ECs of heat shocked *puc-lacZ* midguts (d; right, red) compared to non-heat shocked *puc-lacZ* midguts (c; right red). *upd3* expression was induced in ECs of heat shocked *upd3.1-lacZ* midguts (f; right, red) compared to non-heat shocked *upd3.1-lacZ* midguts (e; right, red).

g-h. Active p38 does not increase in ECs upon high salt stress. p38 activation did not increase in ECs in midguts of w^{1118} fed high salt (0.4M NaCl) diet (h; right, red) compared to midguts of w^{1118} fed a standard diet (g; right, red).

DNA is in blue. Scale bars in a-h are 50 µm. Representative images in a-b, g-h from 3 experiments; c-f, 2 experiments.

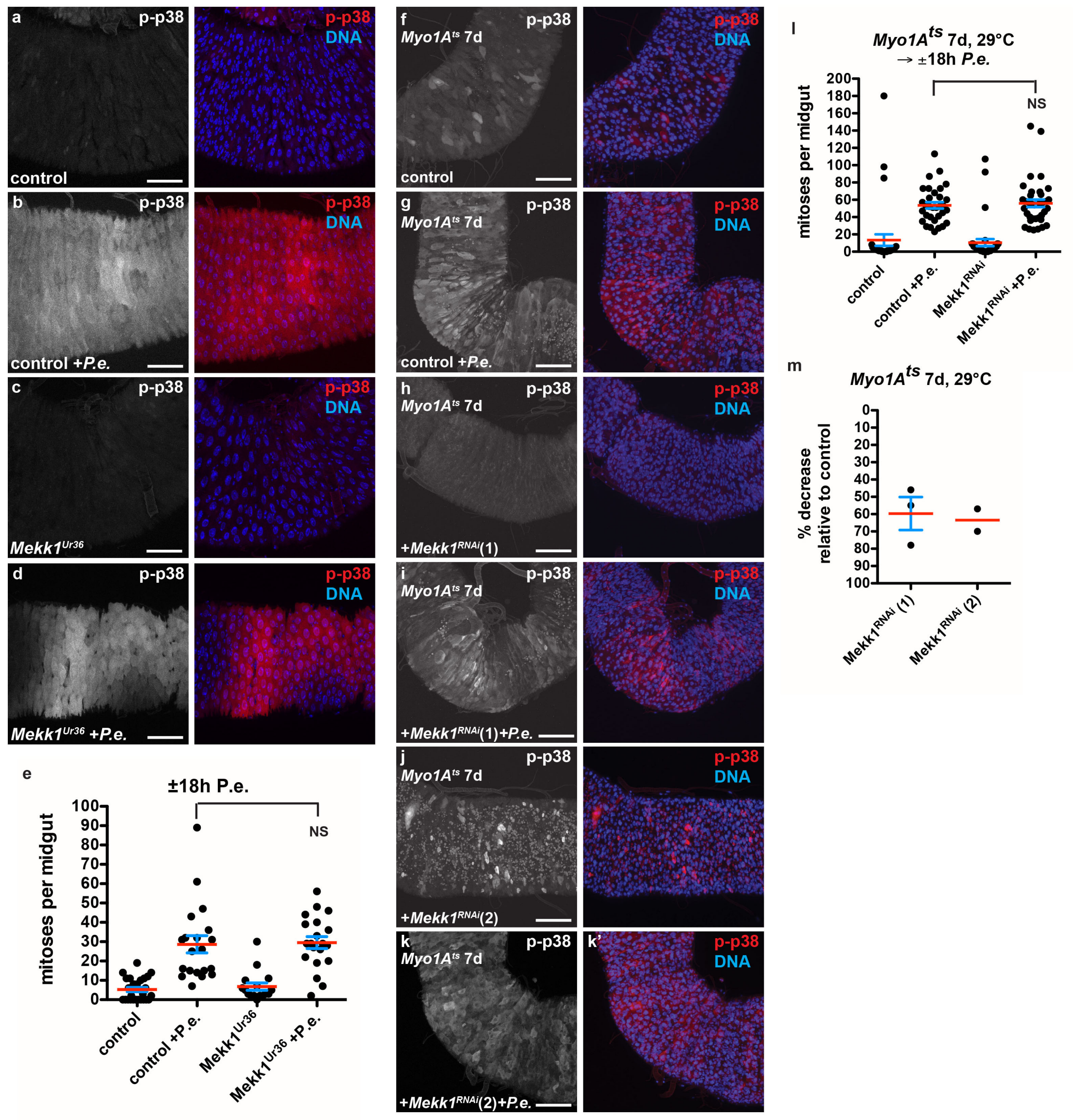

Supplementary Figure 6. SAP3K Mekk1 is not required for p38 activation nor ISC-mediated regeneration upon damage.

a-d. Mekk1 is not required for p38 activation in ECs upon *P.e.* infection. p38 activation was increased in ECs in *P.e.* infected control midguts (b; right, red) compared to uninfected control midguts (a; right, red) or uninfected $Mekk1^{Ur36}$ midguts (c; right, red). p38 activation was increased in ECs of *P.e.* infected $Mekk1^{Ur36}$ midguts (d; right, red) similar to infected control midguts (b; right, red).

e. Mekk1 signaling is not required for ISC-mediated regeneration. ISC proliferation was similar between *P.e.* infected $Mekk1^{Ur36}$ midguts and *P.e.* infected control midguts (Mann-Whitney; P=0.4816). Mean number of phosphorylated histone H3 Ser 10-positive cells per midgut with s.e.m. in uninfected control midguts (n=26 midguts), *P.e.* infected control midguts (n=20 midguts), uninfected $Mekk1^{Ur36}$ midguts (n=16 midguts) and *P.e.* infected $Mekk1^{Ur36}$ midguts (n=20 midguts). Midguts pooled from 2 independent experiments. NS, not significant.

f-k. Mekk1 signaling is not required for p38 activation in ECs upon *P.e.* infection. p38 activation was increased in ECs in *P.e.* infected control (EC-specific; $Myo1A^{ts}$) midguts (g; right, red) compared to uninfected control midguts (f; right, red) or uninfected midguts expressing $Mekk1^{RNAi}$ (1 or 2) in ECs for 7 days with $Myo1A^{ts}$ (h,j; right, red). p38 was activated in *P.e.* infected midguts expressing $Mekk1^{RNAi}$ (1 or 2) in ECs for 7 days with $Myo1A^{ts}$ (i,k; right, red) similar to *P.e.* infected control midguts.

l. Mekk1 signaling is not required in ECs to promote ISC-mediated regeneration. ISC proliferation was similar between *P.e.* infected midguts expressing $Mekk1^{RNAi}$ in ECs for 7 days with $Myo1A^{ts}$ and *P.e.* infected control midguts (P= 0.8899). Mean number of phosphorylated histone H3 Ser 10- positive cells per midgut with s.e.m. in control midguts ($Myo1A^{ts}$) (n=32 midguts), *P.e.* infected control midguts (n=32 midguts), midguts expressing $Mekk1^{RNAi}$ (1) in ECs for 7 days with $Myo1A^{ts}$ (n=35 midguts), and in *P.e.* infected midguts expressing $Mekk1^{RNAi}$ (1) in ECs for 7 days with $Myo1A^{ts}$ (n=37 midguts). Midguts pooled from 2 independent experiments. NS, not significant.

m. *Mekk1* mRNA levels are depleted in midguts expressing $Mekk1^{RNAi}$ (1 and 2) in ECs. Mean % decrease with s.e.m. from 3 ($Mekk1^{RNAi}$ *(1)*) independent experiments or without s.e.m. from 2 ($Mekk1^{RNAi}$ (2)) independent experiments in *Mekk1* mRNA levels determined by qPCR from midguts expressing $Mekk1^{RNAi}$ (1 or 2) in ECs for 7 days with $Myo1A^{ts}$.

DNA is in blue. Scale bars in a-d, f-k are 50 µm. Representative images in a-d from 2 experiments; f-k, 2 experiments. Source data are provided as a Source Data File.

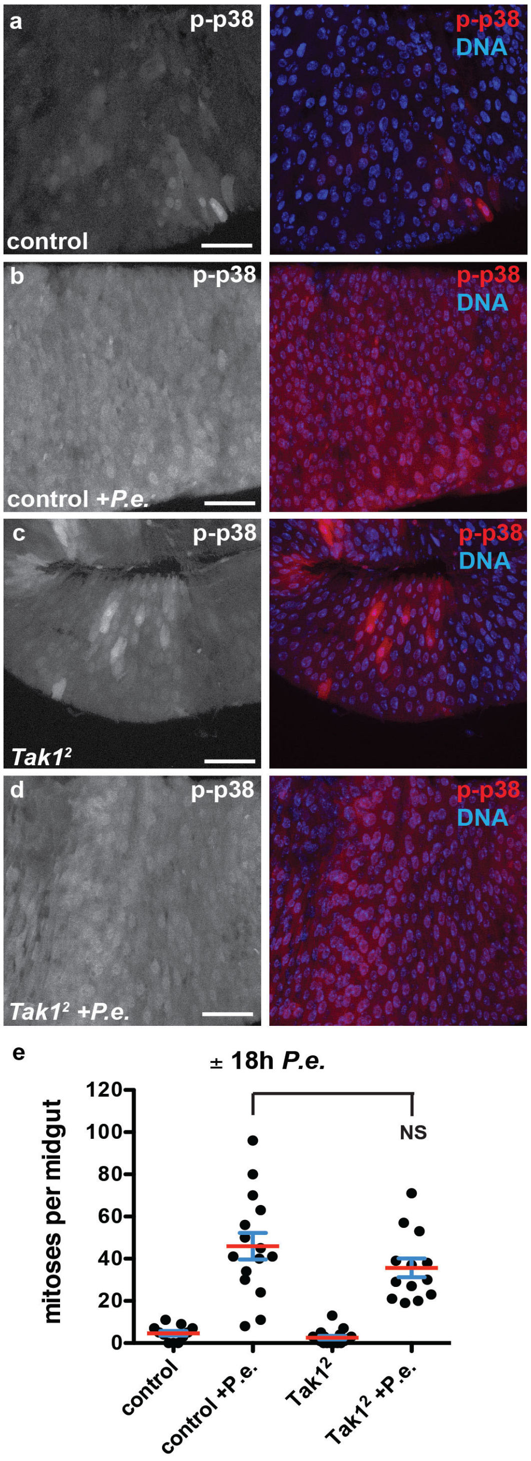

Supplementary Figure 7. SAP3K Tak1 is not required for p38 activation upon infection or ISC-mediated regeneration

a-d. Tak1 is not required for p38 activation in ECs upon *P.e.* infection. p38 activation was increased in ECs of *P.e.* infected control midguts (b; right, red) compared to uninfected control midguts (a; right, red) or uninfected *Tak1*^{2} midguts (c; right, red). Basal active p38 levels were however increased in *Tak1*^{2} midguts (c; right, red) relative to uninfected control midguts (a; right, red). p38 activation was increased in ECs of *P.e.* infected *Tak1*^{2} midguts (d; right, red) similar to infected control midguts (b; right, red).

e. Tak1 signaling is not required for ISC-mediated regeneration. ISC proliferation was similar between *P.e.* infected *Tak1²* midguts and *P.e.* infected control midguts (Mann-Whitney; P=0.1599). Mean number of phosphorylated histone H3 Ser 10-positive cells per midgut with s.e.m. in uninfected control midguts (n=11 midguts), *P.e.* infected control midguts (n=15 midguts), uninfected *Tak1²* midguts (n=17 midguts) and *P.e.* infected *Tak1²* midguts (n=13 midguts). Midguts pooled from 2 independent experiments. NS, not significant.

DNA is in blue. Scale bars in a-d are 50 µm. Representative images in a-d from 2 experiments. Source data are provided as a Source Data File.

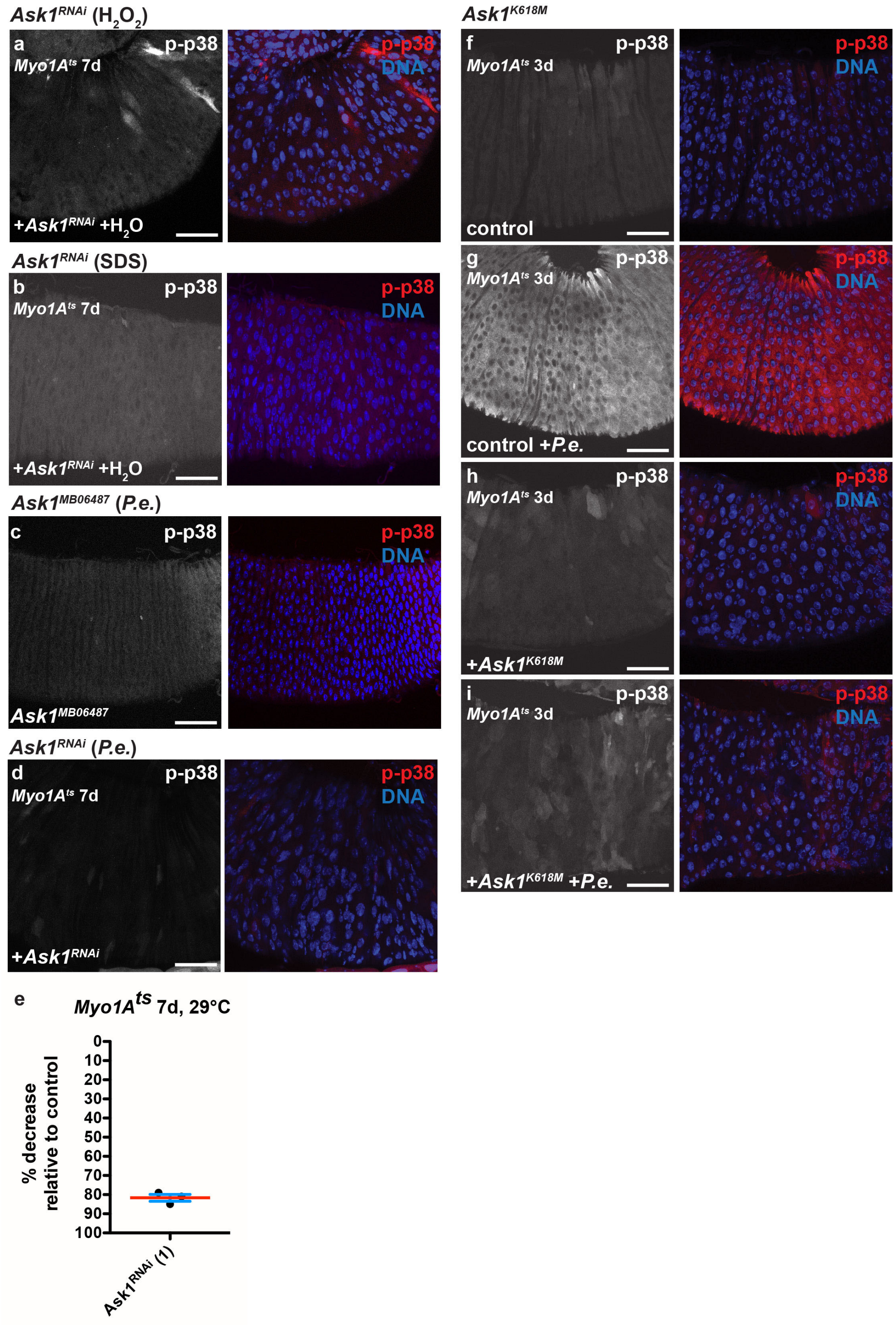

Supplementary Figure 8. Ask1 loss in enterocytes did not affect basal p38 activity

a-d. Basal p38 activity is unaffected upon Ask1 loss in enterocytes. p38 activity is unaffected in H_2O-fed midguts expressing *Ask1*^{RNAi} in ECs for 7 days with *Myo1A*^{ts} (a,b; right, red) for H_2O_2 and SDS- stress experiments (see Figure 4) and in uninfected midguts of *Ask1*^{MB05487} flies and those expressing *Ask1*^{RNAi} (1) in ECs for 7 days (c,d; right, red) for *P.e.* stress experiments (see Figure 5).

e. *Ask1* mRNA levels are depleted in midguts expressing $Ask1^{RNAi}$ (1) in ECs. Mean % decrease with s.e.m from 3 independent experiments in *Ask1* mRNA levels determined by qPCR from midguts expressing $Ask1^{RNAi}$ (1) in ECs for 7 days with $Myo1A^{ts}$.

f-i. Ask1 kinase activity is required for p38 activation in ECs upon *P.e.* infection. p38 activation increased in ECs in *P.e.* infected control ($Myo1A^{ts}$) midguts (g; right, red) compared to uninfected control midguts (f; right, red) or uninfected midguts expressing a kinase dead form of Ask1 ($Ask1^{K618M}$) in ECs for 3 days with $Myo1A^{ts}$ (h; right, red). p38 activation is blocked in *P.e.* infected midguts expressing $Ask1^{K618M}$ in ECs for 3 days with $Myo1A^{ts}$ (i; right, red) compared to *P.e.* infected control midguts.

DNA is in blue. Scale bars in a-i are 50 µm. Representative images in a-b,d from 3 experiments.; c, f-i, 2 experiments. Source data are provided as a Source Data File.

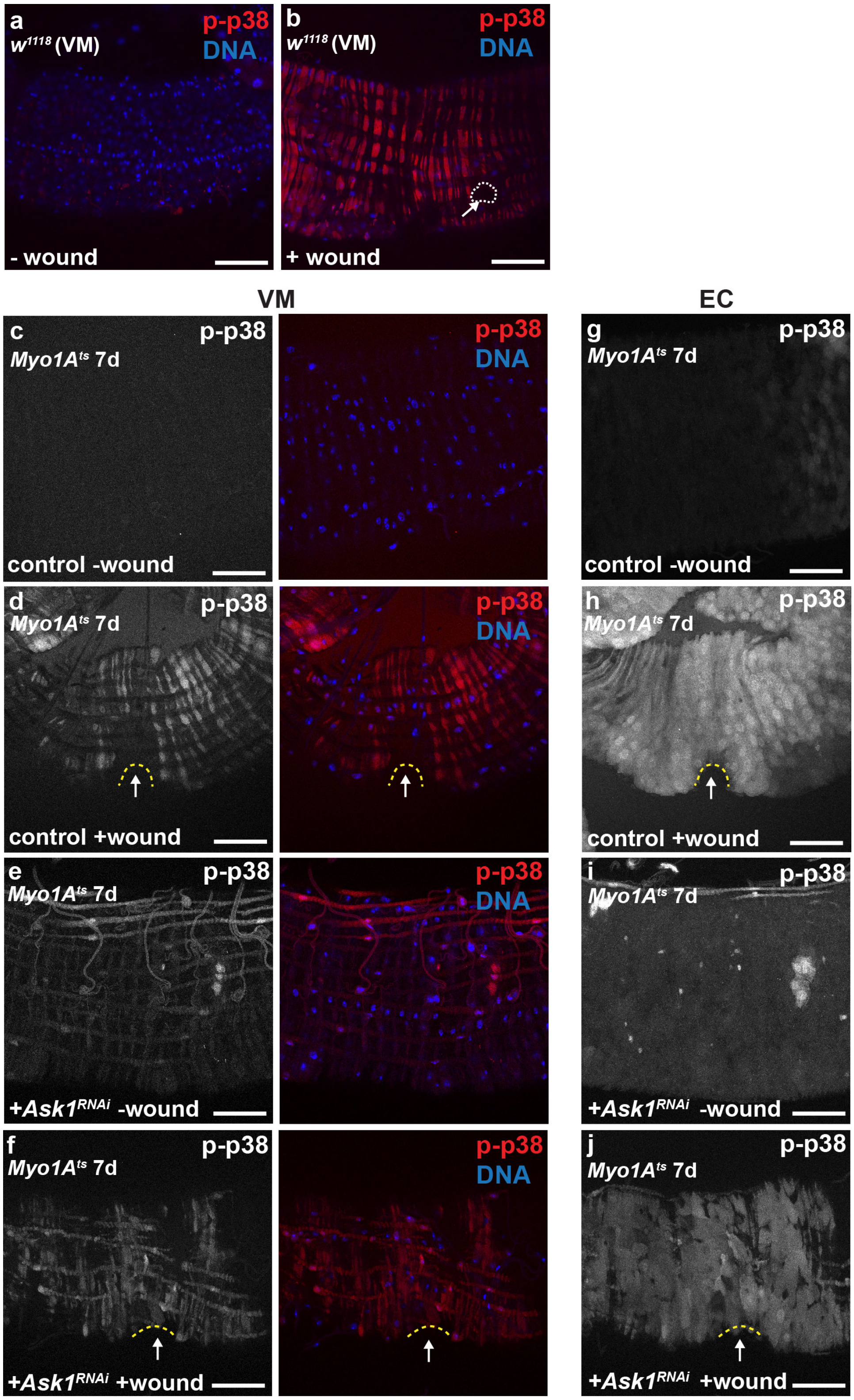

Supplementary Figure 9. Ask1 is required for p38 activation in enterocytes upon wounding

a-b. Wounding with a needle activates p38 signaling in the visceral muscle (VM) (b, red) compared to unwounded midgut VM (a). Wound site is indicated with an arrow and outlined with dashed line.

c-f. p38 activation is increased in VM of wounded midguts (d; right, red) compared to unwounded midguts (c; right, red) and unwounded midguts expressing *Ask1*^{RNAi} in ECs for 7 days with *Myo1A*^{ts} (e; right, red). p38 activation in the VM of wounded midguts expressing *Ask1*^{RNAi} in ECs for 7 days with *Myo1A*^{ts} (f, right, red) is similar to the VM of control wounded midguts.

g-j. p38 activation is increased in ECs of wounded midguts (h) compared to unwounded midguts (g) and unwounded midguts expressing *Ask1*^{RNAi} in ECs for 7 days with *Myo1A*^{ts} (i). p38 activation is decreased in ECs of wounded midguts expressing *Ask1*^{RNAi} in ECs for 7 days with *Myo1A*^{ts} (j) relative to ECs of control wounded midguts.

DNA is in blue. Scale bars in a-h are 50 µm. Representative images in a-h from 2 experiments.

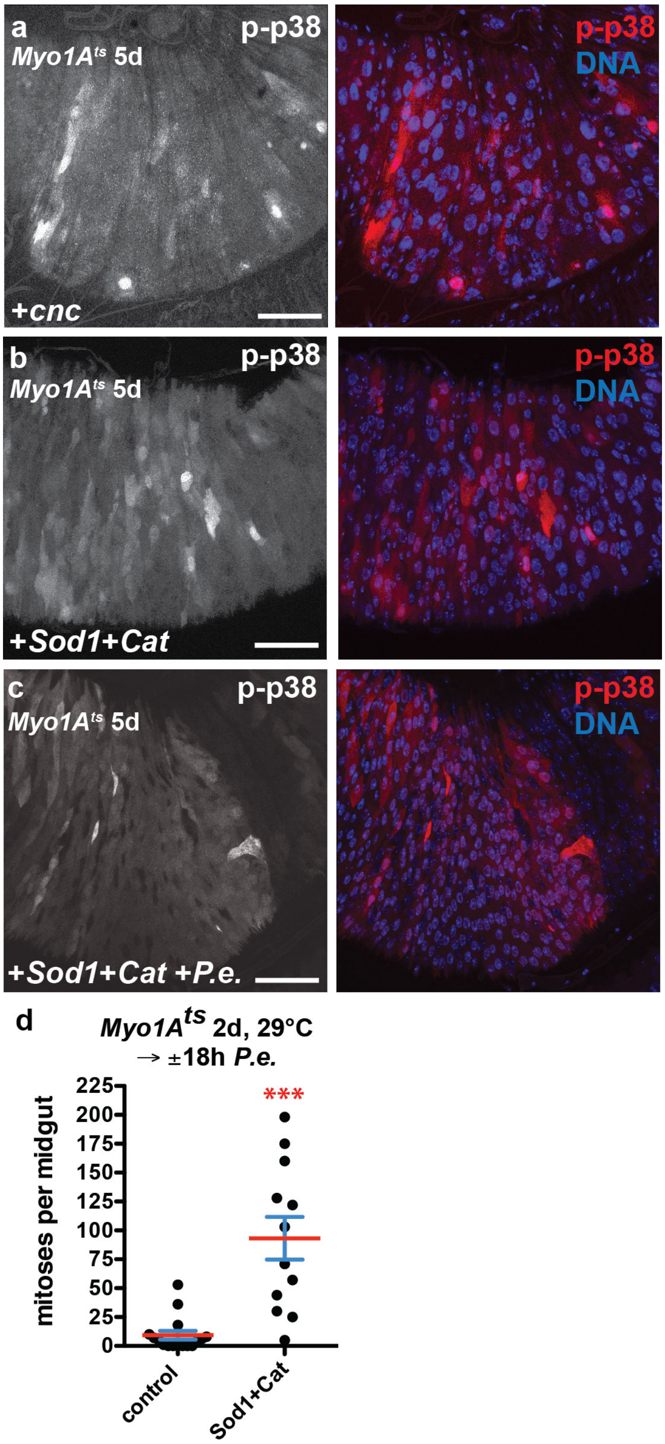

Supplementary Figure 10. Redox stress in enterocytes promotes p38 activation and increases stem cell proliferation

a-b. p38 was mildly activated in midguts overexpressing Nrf2 (cnc) (a; right, red) or co-overexpressing Sod1 and Catalase (b; right, red) in ECs for 5 days with *Myo1A*^{ts}. See Fig. 6 for control.

c. p38 activation is blocked in *P.e.* infected midguts overexpressing Sod1 and Catalase (c; right, red) in ECs for 5 days with *Myo1A*^{ts}.

d. Co-overexpression of Sod1 and Catalase in enterocytes induces ISC proliferation. Mean number of phosphorylated histone H3 Ser 10- positive cells per midgut with s.e.m. in control midguts (*Myo1A*^{ts} alone) (n=16 midguts from 1 experiment) and midguts co-overexpressing *Sod1* and *Catalase* (*Cat*) for 2 days with *Myo1A*^{ts} (n=12 midguts from 1 experiment).

DNA is in blue. Scale bars in a-c are 50 µm. Representative images in a-c from 3 experiments. Source data are provided as a Source Data File.

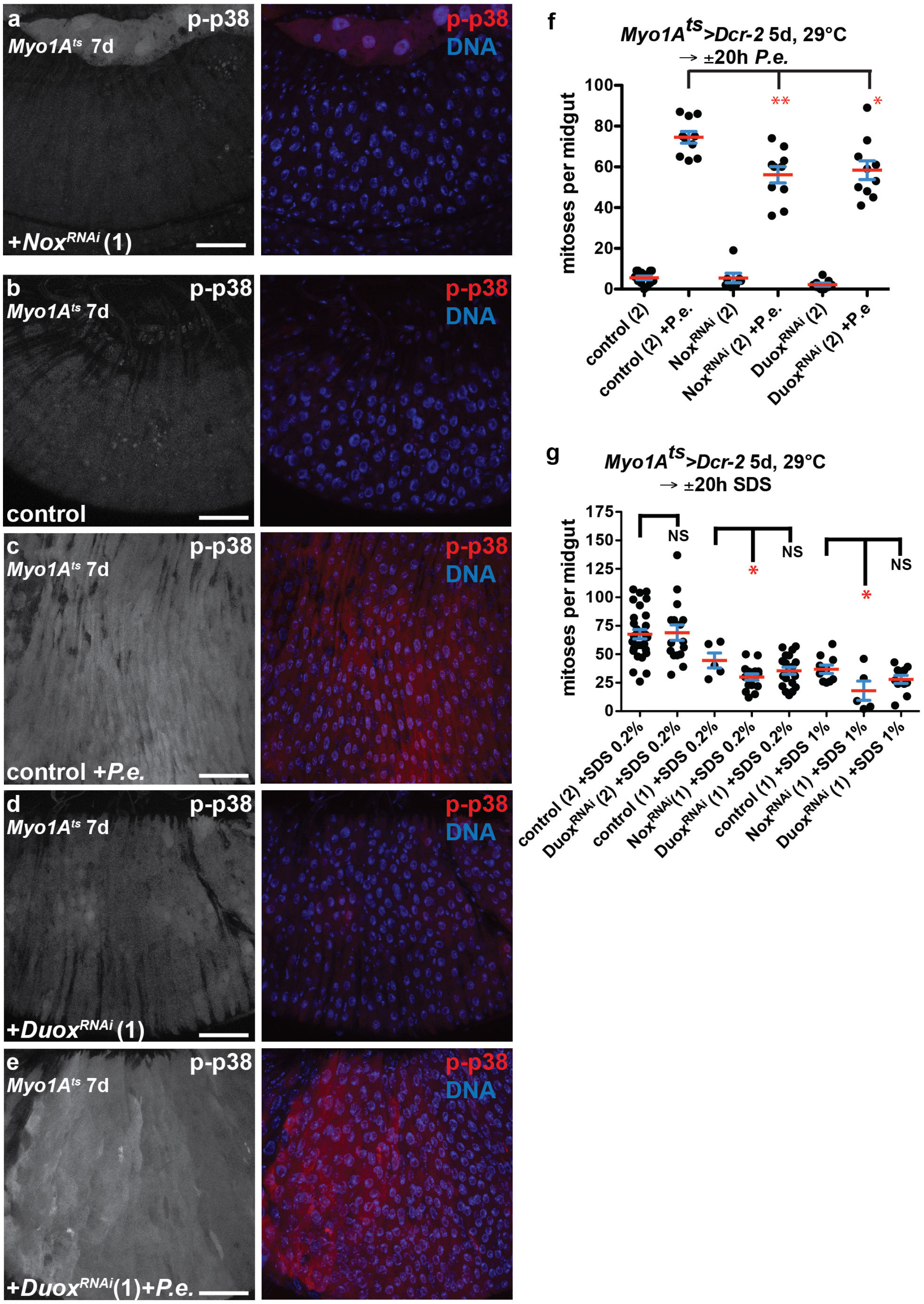

Supplementary Figure 11. Duox-derived ROS in enterocytes promote intestinal regeneration upon pathogenic infection but not upon detergent stress

a. Nox depletion in enterocytes did not affect basal p38 activation. Uninfected midguts expressing *Nox*^{RNAi} in ECs for 7 days with *Myo1A*^{ts} (a; right, red).

b-e. Duox is not required for p38 activation in ECs upon *P.e.* infection. p38 is activated similarly in both *P.e.* infected control midguts (*Myo1A*^{ts}) (c; right, red) and *P.e.* infected midguts expressing *Duox*^{RNAi} in ECs for 7 days with *Myo1A*^{ts} (e; right, red) compared to uninfected control midguts (b; right, red) and midguts expressing *Duox*^{RNAi} in ECs for 7 days with *Myo1A*^{ts} (d; right, red).

f. Nox and Duox are required in ECs for ISC-mediated regeneration upon pathogenic infection. ISC proliferation was blocked in *P.e.* infected midguts expressing *Nox*^{RNAi} (2) or *Duox*^{RNAi} (2) for 5 days with *Myo1A*^{ts}*>Dcr-2* compared to *P.e.* infected control midguts (Mann Whitney test; P=0.0017 (*Nox*^{RNAi} (2); P= 0.0125 (*Duox*^{RNAi} (2)). Mean number of phosphorylated histone H3 Ser 10- positive cells per midgut with s.e.m. in control (2) midguts (n=13 midguts) (*Myo1A*^{ts}*>Dcr-2* alone), *P.e.* infected control (2) midguts (n=10 midguts), midguts expressing *Nox*^{RNAi} (2) in ECs for 5 days with *Myo1A*^{ts}*>Dcr-2* (n=7 midguts), *P.e* infected midguts expressing *Nox*^{RNAi} (2) in ECs for 5 days (n=10 midguts), midguts expressing *Duox*^{RNAi} (2) in ECs for 5 days with *Myo1A*^{ts}*>Dcr-2* (n=9 midguts) and *P.e* infected midguts expressing *Duox*^{RNAi} (2) in ECs for 5 days (n=10 midguts). Midguts from 1 representative experiment.

g. Nox but not Duox is required in ECs for ISC-mediated regeneration upon detergent exposure. ISC proliferation was blocked in SDS 0.2% and SDS 1% fed (20 hours) midguts expressing *Nox*^{RNAi} (1) for 5 days with *Myo1A*^{ts}*>Dcr-2* compared to SDS 0.2% and SDS 1% fed control (1) midguts (SDS 0.2%: unpaired *t*; P=0.0262; SDS 1%: unpaired *t*; P=0.0311). No significant differences were observed in SDS 0.2% and SDS 1% fed (20 hours) midguts expressing *Duox*^{RNAi} (1) for 5 days with *Myo1A*^{ts}*>Dcr-2* compared to SDS 0.2% and SDS 1% fed control (1) midguts (SDS 0.2%: unpaired *t*; P=0.2161; SDS 1%: unpaired *t*; P=0.0923) and between SDS 0.2% fed (20 hours) midguts expressing *Duox*^{RNAi} (2) for 5 days with *Myo1A*^{ts}*>Dcr-2* compared to SDS 0.2% fed control (2) midguts (unpaired t; P=0.8535). Mean number of phosphorylated histone H3 Ser 10- positive cells per midgut with s.e.m. from 2 independent experiments in control (2) midguts (n=29 midguts) and midguts expressing *Duox*^{RNAi} (2) in ECs for 5 days (n=16 midguts) from 0.2% SDS-fed flies; from 1 representative independent experiment in control (1) midguts (n=5 midguts) and midguts expressing *Nox*^{RNAi} (1) in ECs for 5 days (n=16 midguts), midgut expressing *Duox*^{RNAi} (1) (n=19 midguts) from 0.2% SDS-fed flies, and in control (1) midguts (n=10 midguts), midgut expressing *Nox*^{RNAi} (1) in ECs for 5 days (n=5 midguts) and midguts expressing *Duox*^{RNAi} (1) in ECs for 5 days (n=11 midguts) of 1% SDS fed flies.

DNA is in blue. Scale bars in a-e are 50 µm. Representative images in a-e from 3 experiments. Source data are provided as a Source Data File.

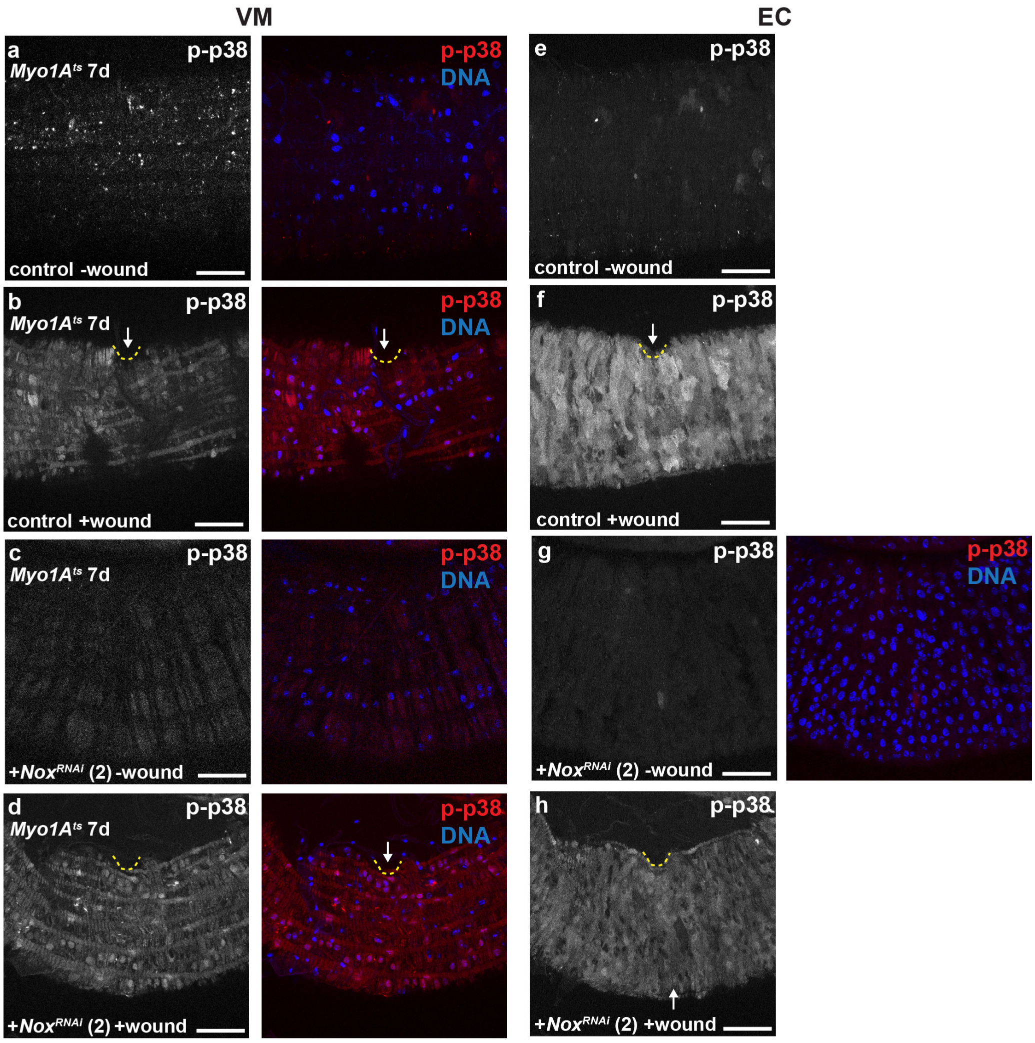

Supplemental Figure 12. Nox-derived ROS promotes p38 activation in enterocytes upon wounding

a-d. Nox-derived ROS is not required in enterocytes to activate p38 in the visceral muscle (VM) upon wounding. p38 activation is increased in the VM of wounded control midguts (b; right, red) compared to the VM of unwounded control midguts (a; right, red) and unwounded midguts expressing Nox^{RNAi} (2) (c; right, red) in ECs for 7 days with $Myo1A^{ts}$. p38 activation is increased in the VM of wounded midguts expressing Nox^{RNAi} (2) in ECs for 7 days with $Myo1A^{ts}$ (d; right, red) similar to wounded control midguts.

e-h. Nox-derived ROS is required to activate p38 in enterocytes after wounding. p38 activation is increased in the ECs of wounded control midguts (f) compared to the ECs of unwounded control midguts (e) and unwounded midguts expressing Nox^{RNAi} (2) (g; right, red) in ECs for 7 days with $Myo1A^{ts}$. p38 activation is blocked in the ECs of wounded midguts expressing Nox^{RNAi} (2) in ECs for 7 days with $Myo1A^{ts}$ (h) compared to wounded control midguts.

DNA is in blue. Scale bars in a-h are 50 µm. Representative images in a-h from 2 experiments.

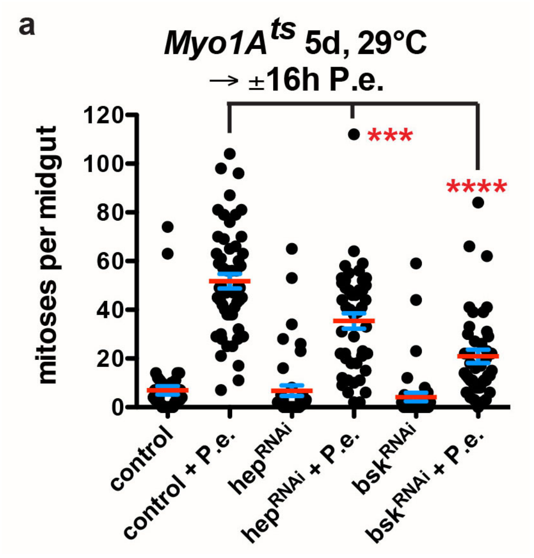

Supplemental Figure 13. JNK activity in enterocytes promotes intestinal regeneration

a. JNK signaling in ECs promotes ISC-mediated intestinal regeneration. The ISC response is inhibited in *P.e.* infected midguts expressing hep^{RNAi} or bsk^{RNAi} in ECs for 5 days with $Myo1A^{ts}$ compared to *P.e.* infected control midguts (Mann-Whitney; P=0.0005, P<0.0001). Mean number of phosphorylated histone H3 Ser 10-positive cells per midgut with s.e.m. in control midguts ($Myo1A^{ts}$) (n=55 midguts), *P.e.* infected control midguts (n=53 midguts), midguts expressing hep^{RNAi} (n=44 midguts) in ECs for 5 days with $Myo1A^{ts}$, *P.e.* infected midguts expressing hep^{RNAi} (n=46 midguts) in ECs for 5 days with $Myo1A^{ts}$, midguts expressing bsk^{RNAi} (n=43 midguts) in ECs for 5 days with $Myo1A^{ts}$ and *P.e.* infected midguts expressing bsk^{RNAi} (n=43 midguts) in ECs for 5 days with $Myo1A^{ts}$. Midguts pooled from 4 independent experiments. Source data are provided as a Source Data File.

Supplementary Methods

Genotypes

Figure 1.

a-c. *w/w*; *Myo1A-GAL4/+*; *tubGAL80^{ts}/+*

d. *w/w*; *Myo1A-GAL4/+*; *tubGAL80^{ts}/+* or

w/w; *Myo1A-GAL4/UAS-p38a^{RNAi}* (1); *tubGAL80^{ts}*/+

e-g *w/w*; *Myo1A-GAL4/+*; *tubGAL80^{ts}/+* or

w/w; *Myo1A-GAL4/+*; *tubGAL80^{ts}/UAS-lic*

h-j. *w/w*; *Myo1A-GAL4/+*; *tubGAL80^{ts}/+* or

w/w; *Myo1A-GAL4/+* ; *tubGAL80^{ts}/UAS-lic^{RNAi}* (1)

Figure 2.

a. *w^{1118}/w^{1118}*

b. *w/w*; *Myo1A-GAL4/+*; *tubGAL80^{ts}/+* or

w/w; *Myo1A-GAL4/UAS-p38a^{RNAi}* (1); *tubGAL80^{ts}*/+ or

w/w; *Myo1A-GAL4/UAS-p38b^{RNAi}* (1); *tubGAL80^{ts}*/+

c. *w*/w; *Myo1A-GAL4/+*; *tubGAL80^{ts}/+* or

w/w; *Myo1A-GAL4/+*; *tubGAL80^{ts}/UAS-lic*

d-g. *w/w*; *Myo1A-GAL4/+*; *tubGAL80^{ts}/10x-STAT-GFP* or

w/w; *Myo1A-GAL4/UAS-p38a^{RNAi}* (1); *tubGAL80^{ts}/10x-STAT-GFP*

g. *w/w*; *Myo1A-GAL4/+*; *tubGAL80^{ts}/10x-STAT-GFP* or

w/w; *Myo1A-GAL4/UAS-p38a^{RNAi}* (1); *tubGAL80^{ts}/10x-STAT-GFP*

Figure 3.

a-b. w^{1118}/w^{1118}

c. *w/w*; *esg-GAL4/*+; *tubGAL80^{ts} UAS-GFP/+*

d. *w/w*; *esg-GAL4/*+; *tubGAL80^{ts} UAS-GFP/UAS-Notch^{RNAi}*

e. *w/w*; *Myo1A-GAL4/+*; *tubGAL80^{ts} UAS-GFP/+*

f. *w/w; Myo1A-GAL4/UAS-mys^{RNAi}; tubGAL80^{ts}/+*

Figure 4.

a-b. *w/w*; *Myo1A-GAL4/+*; *tubGAL80^{ts} UAS-GFP/+*

c. *w/w*; *Myo1A-GAL4/+*; *tubGAL80^{ts}/UAS-Ask1^{RNAi}*

d-e. *w/w*; *Myo1A-GAL4/+*; *tubGAL80^{ts} UAS-GFP/+*

f. *w/w*; *Myo1A-GAL4/+*; *tubGAL80^{ts}/UAS-Ask1^{RNAi}*

Figure 5.

a-b. w^{1118}

c. *w^{1118}; Ask1^{MB06487}*

d-e. *w*/w; *Myo1A-GAL4/+*; *tubGAL80^{ts}/+*

f. *w/w*; *Myo1A-GAL4/+*; *tubGAL80^{ts}/UAS-Ask1^{RNAi}*

g. *w/w*; *Myo1A-GAL4/+*; *tubGAL80^{ts}/+*

or *w/w*; *Myo1A-GAL4/+*; *tubGAL80^{ts}/UAS-Ask1^{RNAi}*

h-i. *w*/w; *Myo1A-GAL4/+*; *tubGAL80^{ts}/+*

j-k. *w/w*; *Myo1A-GAL4/+*; *tubGAL80^{ts}/UAS-Ask1^{RNAi}*

Figure 6.

a-b. *w/w*; *Myo1A-GAL4/+*; *tubGAL80^{ts}/+*

c. *w/w*; *Myo1A-GAL4/+*; *tubGAL80^{ts}/UAS-Cnc*

d. *w/w*; *Myo1A-GAL4/+*; *tubGAL80^{ts}/+*

or *w/w*; *Myo1A-GAL4/+*; *tubGAL80^{ts}/UAS-Cnc*

e. *w UAS-Dcr-2/w*; *Myo1A-GAL4/+*; *tubGAL80^{ts}/+*

or *w UAS-Dcr-2/w*; *Myo1A-GAL4/+*; *tubGAL80^{ts}/UAS-Cnc*

f. *w^{1118}/w^{1118}*

Figure 7.

a-b. *w/w*; *Myo1A-GAL4/+*; *tubGAL80^{ts}/+*

c. *w/w*; *Myo1A-GAL4/+*; *tubGAL80^{ts}/UAS-Nox^{RNAi}* (1)

d. *w UAS-Dcr-2/w*; *Myo1A-GAL4/+*; *tubGAL80^{ts}/+*

or *w UAS-Dcr-2/w*; *Myo1A-GAL4/+*; *tubGAL80^{ts}/UAS-Nox^{RNAi}* (1)

or *w UAS-Dcr-2/w*; *Myo1A-GAL4/+*; *tubGAL80^{ts}/UAS-Duox^{RNAi}* (1)

e. *w UAS-Dcr-2/w*; *Myo1A-GAL4/+*; *tubGAL80^{ts}/+*

or *w UAS-Dcr-2/*w; *Myo1A-GAL4/UAS-Nox^{RNAi}* (2); *tubGAL80^{ts}/+*

f-g. *w/w*; *Myo1A-GAL4/+*; *tubGAL80^{ts}/+*

h. *w/w*; *Myo1A-GAL4/UAS-Nox^{RNAi}* (2); *tubGAL80^{ts}/+*

Supplementary Figure 1.

a-b. *w/w; Myo1A-GAL4/UAS-p38a^{RNAi}* (1)*; tubGAL80^{ts}/+*

c-d. *w/w*; *esg-GAL4 tubGAL80^{ts}*; *UAS-Flp, act<CD2>GAL4, UAS-GFP*

e-f. *w/w*; *tubGAL80^{ts} UAS-GFP/+*; *how(24B)-GAL4/+*

g. *w/w*; *tubGAL80^{ts} UAS-GFP/+*; *how(24B)-GAL4/+* or

w/w; *tubGAL80^{ts} UAS-GFP/UAS-p38a^{RNAi}* (1); *how(24B)-GAL4/+*

h. *w/w; Myo1A-GAL4/+ tubGAL80^{ts}/+*

or *w/w; Myo1A-GAL4/UAS-p38a^{RNAi}* (2)*; tubGAL80^{ts}/+*

i. *w/w; Myo1A-GAL4/UAS-p38a^{RNAi}* (1)*; tubGAL80^{ts}/10x Stat-GFP*

j. *w/w*; *Myo1A-GAL4/+*; *tubGAL80^{ts}/+* or

w/w; Myo1A-GAL4/UAS-p38a^{RNAi} (1)*; tubGAL80^{ts}/+* or

w/w; Myo1A-GAL4/UAS-p38a^{RNAi} (2)*; tubGAL80^{ts}/+*

Supplementary Figure 2.

a-b. *w/w*; *Myo1A-GAL4/+*; *tubGAL80^{ts}/+*

c-d. *w/w*; *Myo1A-GAL4/UAS-p38b^{RNAi}* (1); *tubGAL80^{ts}/+*

e. *w/w*; *Myo1A-GAL4/+*; *tubGAL80^{ts}/+*

or *w/w*; *Myo1A-GAL4/UAS-p38a^{RNAi}*; *tubGAL80^{ts}/+*

or *w/w*; *Myo1A-GAL4/UAS-p38b^{RNAi}* (1); *tubGAL80^{ts}/+*

f. *w/w*; *Myo1A-GAL4/+*; *tubGAL80^{ts}/+*

or *w/w*; *Myo1A-GAL4/UAS-p38a^{RNAi}* (1 or 2); *tubGAL80^{ts}/+*

g. *w/w*; *Myo1A-GAL4/+*; *tubGAL80^{ts}/+*

or *w/w*; *Myo1A-GAL4/UAS-p38b^{RNAi}* (1); *tubGAL80^{ts}/+*

or *w UAS-p38b^{antisense}/w*; *Myo1A-GAL4/+*; *tubGAL80^{ts}/+*

or *w UAS-p38^{DN}*/w; *Myo1A-GAL4/+*; *tubGAL80^{ts}/+*

h. *w^{1118}/w^{1118}* or *w^{1118}/w^{1118}* ; *p38a^{1}/+* or *w^{1118}/w^{1118}*; *p38a^{1}/p38a^{1}*

i. *w^{1118}/w^{1118}* or *w^{1118}/w^{1118}*; *p38b^{ex9}/+* or *w^{1118}/w^{1118}*; *p38b^{ex9}/p38b^{ex9}*

Supplementary Figure 3.

a. *w/w*; *Myo1A-GAL4/+*; *tubGAL80*^{ts}*/+*

b. *w/w*; *Myo1A-GAL4/+*; *tubGAL80*^{ts}*/UAS-lic*

c. *w/w*; *Myo1A-GAL4/+*; *tubGAL80*^{ts}*/+* or

w/w; *Myo1A-GAL4/+*; *tubGAL80*^{ts}*/UAS-lic*

d. *w/w*; *Myo1A-GAL4/+*; *tubGAL80*^{ts}*/UAS-lic*^{RNAi} (1)

e. *w/w*; *Myo1A-GAL4/+*; *tubGAL80*^{ts}*/+*

or *w/w*; *Myo1A-GAL4/+*; *tubGAL80*^{ts}*/UAS-lic*^{RNAi}

f-g. *w/w*; *Myo1A-GAL4/+*; *tubGAL80*^{ts}*/+*

h-i. *w/w*; *Myo1A-GAL4/UAS-lic*^{RNAi} (2); *tubGAL80*^{ts}/+

Supplementary Figure 4.

a. *w*^{1118}*/w*^{1118} or *w*^{1118}*/w*^{1118}; *Atf2*^{PB}*/Atf2*^{PB}

b. *w/w*; *Myo1A-GAL4/+*; *tubGAL80*^{ts}*/+*

or *w/w*; *Myo1A-GAL4/+*; *tubGAL80*^{ts}*/UAS-Atf2*^{RNAi} (1)

or *w/w*; *Myo1A-GAL4/+*; *tubGAL80*^{ts}*/UAS-Atf2*^{RNAi} (2)

c. *yw MK2*$^{\Delta 1A}$*/yw MK2*$^{\Delta 1A}$ and *yw MK2*$^{\Delta 43}$*/yw MK2*$^{\Delta 43}$

d. *w*^{1118}*/w*^{1118} or *w*^{1118}*/w*^{1118}; *Atf2*^{PB}*/Atf2*^{PB}

Supplementary Figure 5.

a-b. *w*^{1118}*/w*^{1118}

c-d. *w*^{1118}*/+*; *+/+*; *puc*^{E69}*/+*

e-f. *w*^{1118}*/w*; *+/+*; *upd3.1-lacZ/+*

g-h. *w*^{1118}*/w*^{1118}

Supplementary Figure 6.

a-b. w^{1118}/w^{1118}

c-d. w^{1118}/w^{1118}; *Mekk1*^{Ur36}/*Mekk1*^{Ur36}

e. w^{1118}/w^{1118} or w^{1118}/w^{1118}; *Mekk1*^{Ur36}/*Mekk1*^{Ur36}

f-g. *w/w*; *Myo1A-GAL4/+*; *tubGAL80*^{ts}*/+*

h-i. *w/w*; *Myo1A-GAL4/+*; *tubGAL80*^{ts}*/UAS-Mekk1*^{RNAi} (1)

j-k. *w*/w; *Myo1A-GAL4/UAS-Mekk1*^{RNAi} (2); *tubGAL80*^{ts}*/+*

l. *w/w*; *Myo1A-GAL4/+*; *tubGAL80*^{ts}*/+*

or *w/w*; *Myo1A-GAL4/UAS-Mekk1*^{RNAi} (2); *tubGAL80*^{ts}/+

m. *w/w*; *Myo1A-GAL4/+*; *tubGAL80*^{ts}*/+*

or *w/w*; *Myo1A-GAL4/+*; *tubGAL80*^{ts}*/UAS-Mekk1*^{RNAi} (1)

or *w/w*; *Myo1A-GAL4/UAS-Mekk1*^{RNAi} (2); *tubGAL80*^{ts}/+

Supplementary Figure 7.

a-b. yw/yw

c-d. yw/yw; Tak1^{2}/Tak1^{2}

e. +/+ or +/+; Tak1^{2}/Tak1^{2}

Supplementary Figure 8.

a-b. *w*/w; *Myo1A-GAL4/+*; *tubGAL80*^{ts}*/UAS-Ask1*^{RNAi}

c. *w/w*; *Ask1*^{MB06487}*/Ask1*^{MB06487}

d. *w*/w; *Myo1A-GAL4/+*; *tubGAL80*^{ts}*/UAS-Ask1*^{RNAi}

e. *w/w*; *Myo1A-GAL4/+*; *tubGAL80*^{ts}*/+*

or *w/w*; *Myo1A-GAL4/+*; *tubGAL80^{ts}/UAS-Ask1^{RNAi}*

f-g. *w/w*; *Myo1A-GAL4/+*; *tubGAL80^{ts}/+*

h-l. *w/w*; *Myo1A-GAL4/UAS-Ask1^{K618M}*; *tubGAL80^{ts}/+*

Supplementary Figure 9.

a-b. *w^{1118}/w^{1118}*

c-d. *w/w*; *Myo1A-GAL4/+*; *tubGAL80^{ts}/+*

e-f. *w/w*; *Myo1A-GAL4/+*; *tubGAL80^{ts}/UAS-Ask1^{RNAi}*

g-h. *w/w*; *Myo1A-GAL4/+*; *tubGAL80^{ts}/+*

i-j. *w/w*; *Myo1A-GAL4/+*; *tubGAL80^{ts}/UAS-Ask1^{RNAi}*

Supplementary Figure 10.

a. *w/w*; *Myo1A-GAL4/+*; *tubGAL80^{ts}/UAS-Cnc*

b-c. *w/w*; *Myo1A-GAL4/UAS-sod1 UAS-catalase*; *tubGAL80^{ts}/+*

d. *w/w*; *Myo1A-GAL4/+*; *tubGAL80^{ts}/+*

or *w/w*; *Myo1A-GAL4/UAS-sod1 UAS-catalase*; *tubGAL80^{ts}/+*

Supplementary Figure 11.

a. *w/w*; *Myo1A-GAL4/+*; *tubGAL80^{ts}/UAS-Nox^{RNAi}*(1)

b-c. *w/w*; *Myo1A-GAL4/+*; *tubGAL80^{ts}/+*

d-e. *w/w*; *Myo1A-GAL4/+*; *tubGAL80^{ts}/UAS-Duox^{RNAi}* (1)

f. *w UAS-Dcr-2/w*; *Myo1A-GAL4/+; tubGAL80^{ts}/+*

or *w UAS-Dcr-2/w*; *Myo1A-GAL4/UAS-Nox^{RNAi}*(2)*; tubGAL80^{ts}/+*

or *w UAS-Dcr-2/w*; *Myo1A-GAL4/UAS-Duox^{RNAi}* (2)*+; tubGAL80^{ts}/+*

g. *w UAS-Dcr-2/w*; *Myo1A-GAL4/+; tubGAL80*^{ts}*/+*

or *w UAS-Dcr-2/w*; *Myo1A-GAL4/+; tubGAL80*^{ts}*/UAS-Nox*^{RNAi}(1)

or *w UAS-Dcr-2/w*; *Myo1A-GAL4/+; tubGAL80*^{ts}*/UAS-Duox*^{RNAi} (1)

or *w UAS-Dcr-2/w*; *Myo1A-GAL4/UAS-Duox*^{RNAi} (2)*; tubGAL80*^{ts}*/+*

Supplementary Figure 12.

a-b. *w/w*; *Myo1A-GAL4/+; tubGAL80*^{ts}*/+*

c-d. *w UAS-Dcr-2/w*; *Myo1A-GAL4/UAS-Nox*^{RNAi}(2)*; tubGAL80*^{ts}*/+*

e-f. *w/w*; *Myo1A-GAL4/+; tubGAL80*^{ts}*/+*

g-h. *w UAS-Dcr-2/w*; *Myo1A-GAL4/UAS-Nox*^{RNAi}(2)*; tubGAL80*^{ts}*/+*

Supplementary Figure 13.

a. *w/w; Myo1A-GAL4/+; tubGAL80*^{ts}*/+*

or w/w*; Myo1A-GAL4/UAS-hep*^{RNAi}*; tubGAL80*^{ts}*/+*

or w/w*; Myo1A-GAL4/UAS-bsk*^{RNAi}*; tubGAL80*^{ts}*/+*
