# Supplementary material for: Damage sensing by a Nox-Ask1-MKK3-p38 signaling pathway mediates regeneration in the adult Drosophila midgut
Source: Nat Commun. 2019 Sep 25;10:4365. doi: 10.1038/s41467-019-12336-w (PMC6761285; doi:10.1038/s41467-019-12336-w)
Supplement: Supplementary file 2 — Reporting Summary [file 41467_2019_12336_MOESM2_ESM.pdf]

nature research

Corresponding author(s): Parthive H. Patel
Bruce A. Edgar

Last updated by author(s): Jul 16, 2019

Reporting Summary

Nature Research wishes to improve the reproducibility of the work that we publish. This form provides structure for consistency and transparency in reporting. For further information on Nature Research policies, see Authors & Referees and the Editorial Policy Checklist.

Statistics

For all statistical analyses, confirm that the following items are present in the figure legend, table legend, main text, or Methods section.

n/a	Confirmed	
☐	☒	The exact sample size (*n*) for each experimental group/condition, given as a discrete number and unit of measurement
☐	☒	A statement on whether measurements were taken from distinct samples or whether the same sample was measured repeatedly
☐	☒	The statistical test(s) used AND whether they are one- or two-sided<br>*Only common tests should be described solely by name; describe more complex techniques in the Methods section.*
☒	☐	A description of all covariates tested
☐	☒	A description of any assumptions or corrections, such as tests of normality and adjustment for multiple comparisons
☐	☒	A full description of the statistical parameters including central tendency (e.g. means) or other basic estimates (e.g. regression coefficient) AND variation (e.g. standard deviation) or associated estimates of uncertainty (e.g. confidence intervals)
☐	☒	For null hypothesis testing, the test statistic (e.g. *F*, *t*, *r*) with confidence intervals, effect sizes, degrees of freedom and *P* value noted<br>*Give P values as exact values whenever suitable.*
☒	☐	For Bayesian analysis, information on the choice of priors and Markov chain Monte Carlo settings
☒	☐	For hierarchical and complex designs, identification of the appropriate level for tests and full reporting of outcomes
☒	☐	Estimates of effect sizes (e.g. Cohen's *d*, Pearson's *r*), indicating how they were calculated

Our web collection on statistics for biologists contains articles on many of the points above.

Software and code

Policy information about availability of computer code

Data collection	Confocal images were acquired with a Leica SP8 confocal microscope using the Leica Application Suite X (2.01.14392) and quantitative real-time PCR data was collected on an Applied Biosystems Step One Plus real-time PCR System using the Applied Biosystems StepOne software (v.2.0) and on a CFX Touch Real-Time system (BioRad) using the CFX manager software v3.1.
Data analysis	All statistical analyses were performed using GraphPad Prism 5 and 8. Quantitative real-time PCR data was analyzed on an Applied Biosystems Step One Plus real-time PCR System using the Applied Biosystems StepOne software (v.2.0). ImageJ 2.0.0-rc-43/1.51p was used to measure the surface area and signal integrated density from maximal intensity projections of confocal images from the enterocyte layer of midguts.

For manuscripts utilizing custom algorithms or software that are central to the research but not yet described in published literature, software must be made available to editors/reviewers. We strongly encourage code deposition in a community repository (e.g. GitHub). See the Nature Research guidelines for submitting code & software for further information.

Data

Policy information about availability of data

All manuscripts must include a data availability statement. This statement should provide the following information, where applicable:

- Accession codes, unique identifiers, or web links for publicly available datasets
- A list of figures that have associated raw data
- A description of any restrictions on data availability

All data are available from the corresponding authors upon request.

Field-specific reporting

Please select the one below that is the best fit for your research. If you are not sure, read the appropriate sections before making your selection.

☒ Life sciences ☐ Behavioural & social sciences ☐ Ecological, evolutionary & environmental sciences

For a reference copy of the document with all sections, see nature.com/documents/nr-reporting-summary-flat.pdf

Life sciences study design

All studies must disclose on these points even when the disclosure is negative.

Sample size	No statistical methods were used to predetermine sample size. Sample sizes for experiments were similar to those found in the field, typically about 5-20 midguts per experiment.
Data exclusions	No data were excluded from analysis.
Replication	Each experiment has been replicated two or more times unless stated otherwise.
Randomization	Animals were selected for their genotype and then randomly chosen to be used for an experiment. The genotype of the samples were concealed and samples were randomly analyzed for imaging or to determine mitoses per midgut.
Blinding	The investigator was blinded during imaging and the quantification of mitoses per midgut by concealing the genotype on the slide. Slides were then randomly analyzed and the genotype was revealed only after the analysis was completed.

Reporting for specific materials, systems and methods

We require information from authors about some types of materials, experimental systems and methods used in many studies. Here, indicate whether each material, system or method listed is relevant to your study. If you are not sure if a list item applies to your research, read the appropriate section before selecting a response.

Materials & experimental systems

n/a	Involved in the study	
☐	☒	Antibodies
☒	☐	Eukaryotic cell lines
☒	☐	Palaeontology
☐	☒	Animals and other organisms
☒	☐	Human research participants
☐	☐	Clinical data

Methods

n/a	Involved in the study	
☒	☐	ChIP-seq
☒	☐	Flow cytometry
☒	☐	MRI-based neuroimaging

Antibodies

Antibodies used	rabbit polyclonal anti-phospho p38 (Cell Signaling), rabbit polyclonal anti-phospho Ser10 histone 3 (Upstate Biotechnology/Millipore), chicken polyclonal anti-β-galactosidase (Abcam) and chicken polyclonal anti-GFP (Life Technologies). Catalog numbers and dilutions can be found in the manuscript.
Validation	rabbit polyclonal anti-phospho p38 (Cell Signaling, #9211) is stated by the manufacturer to react with Drosophila melanogaster phosphorylated p38. Nevertheless, we have validated the antibody ourselves by depleting both phosphorylatable forms of fly p38 and detecting low phospho-p38 levels upon stress (phospho-p38 levels increase with stress). Rabbit polyclonal anti-phospho Ser10 histone 3 (Upstate Biotechnology/Millipore, #06-570), chicken polyclonal anti-β-galactosidase (Abcam, #ab9361) and chicken polyclonal anti-GFP (Life Technologies, #A10262) have been previously used in Patel et al., 2015 (Nature Cell Biology).

Animals and other organisms

Policy information about studies involving animals; ARRIVE guidelines recommended for reporting animal research

Laboratory animals	Drosophila melanogaster, female, 5-10 days old
Wild animals	n/a
Field-collected samples	n/a
Ethics oversight	No ethical approval or guidance was required for Drosophila work

Note that full information on the approval of the study protocol must also be provided in the manuscript.

Clinical data

Policy information about clinical studies

All manuscripts should comply with the ICMJE guidelines for publication of clinical research and a completed CONSORT checklist must be included with all submissions.

Clinical trial registration	n/a
Study protocol	n/a
Data collection	n/a
Outcomes	n/a
